# Supplementary material for: TAxonomy of Self-reported Sedentary behaviour Tools (TASST) framework for development, comparison and evaluation of self-report tools: content analysis and systematic review
Source: BMJ Open. 2017 Apr 8;7(4):e013844. doi: 10.1136/bmjopen-2016-013844 (PMC5775464; doi:10.1136/bmjopen-2016-013844)
Supplement: Author's manuscript [file bmjopen-2016-013844.draft_revisions.pdf]

# TAxonomy of Self-reported Sedentary behaviour Tools (TASST): a framework for development, comparison and evaluation

| | |
|---|---|
| Journal: | *BMJ Open* |
| Manuscript ID | bmjopen-2016-013844 |
| Article Type: | Research |
| Date Submitted by the Author: | 10-Aug-2016 |
| Complete List of Authors: | Dall, Philippa; Glasgow Caledonian University School of Health and Life Sciences, Department of Psychology, Social Work & Allied Health Sciences<br>Coulter, Elaine; University of Glasgow<br>Fitzsimons, Claire F.; Univ Edinburgh, ISPEHS<br>Skelton, Dawn; Glasgow Caledonian University, School of Health & Life Sciences<br>Chastin, Sebastian; Glasgow Caledonian University |
| <b>Primary Subject Heading</b>: | Public health |
| Secondary Subject Heading: | Epidemiology, Research methods, Sports and exercise medicine |
| Keywords: | sedentary behaviour, sitting, population surveillance, measurement, validation |

SCHOLARONE™ Manuscripts

**Title:** TAxonomy of Self-reported Sedentary behaviour Tools (TASST): a framework for development, comparison and evaluation

**Authors:**

Dall PM[1], Coulter EH[2], Fitzsimons CF[3], Skelton DA[1], Chastin SFM[1], on behalf of the Seniors USP Team.

[1] Institute for Applied Health Research, Glasgow Caledonian University, Cowcaddens Road, Glasgow, G4 0BA

[2] Department of Nursing and Healthcare, Glasgow University, 59 Oakfield Avenue, Glasgow, G12 8LL

[3] Sport, Physical Education and Health Sciences Institute, Edinburgh University, St Leonard's Land, Holyrood Road, Edinburgh, EH8 8AQ

corresponding author:

Dr Philippa Dall

School of Health & Life Sciences

Glasgow Caledonian University

Cowcaddens Road

Glasgow

G4 0BA

philippa.dall@gcu.ac.uk

Word count: 4,746

For peer review only

**ABSTRACT**

**Objective:** Sedentary behaviour (SB) has distinct deleterious health outcomes, yet there is no consensus on best practice for measurement. This study aimed to identify the optimal tool for population surveillance of SB, using a systematic framework.

**Design:** A framework, TAxonomy of Self-report SB Tools (TASST), was developed based on a systematic inventory of existing tools. The inventory was achieved through a systematic review of studies reporting SB and tracing back to the original description. A systematic review of the accuracy and sensitivity to change of these tools was then mapped against TASST domains.

**Data Sources:** Systematic searches were conducted via EBSCO.

**Eligibility Criteria for selecting studies:** The inventory included tools measuring SB in adults that could be self-completed at one sitting, and excluded tools measuring SB in specific populations or contexts. The systematic review included studies reporting on the accuracy against an objective measure of SB and/or sensitivity to change of a tool in the inventory.

**Results:** The systematic review identified 32 distinct tools comprising 141 questions. The TASST framework has four domains (type of assessment, recall period, temporal unit, and assessment period), which characterised all self-report SB tools. Fourteen studies evaluated accuracy and/or sensitivity to change representing only 6 taxa. Assessing SB as a sum of behaviours and using a previous day recall were the most promising features of existing tools. Accuracy was poor for all existing tools, with both under and over estimation of SB. There was a complete lack of evidence about sensitivity to change.

**Conclusions:** Despite the limited evidence, mapping existing SB tools onto the TASST framework has enabled informed recommendations to be made about the most promising features for a surveillance tool, and to identify the aspects on which future research and development of SB surveillance tools should focus.

For peer review only - http://bmjopen.bmj.com/site/about/guidelines.xhtml

**Systematic Review Registration**

PROSPERO (CRD42014009851)

**KEY WORDS:**

sedentary behaviour; sitting; population surveillance; measurement; validation

**STRENGTHS AND LIMITATIONS OF THIS STUDY:**

- A systematic approach was taken towards classifying self-reported measures of sedentary behaviour, allowing a structured approach to measurement in the future
- An example of use of the framework is presented, mapping accuracy and sensitivity to change of self-report sedentary behaviour measures on to the framework
- Although designed to be generic, the TASST framework was developed excluding tools measuring sedentary behaviour in specialised contexts, e.g. children, and the framework may therefore not cover some aspects of these tools
- There is the potential for a language bias, as full-text articles notm in English were not included in the systematic reviews.

For peer review only

## BACKGROUND

Physical inactivity is currently at pandemic levels [1] and is a global public health concern. Sedentary behaviour (SB), an umbrella term for all waking time spent in non-exercising sitting or reclining postures [2, 3] such as sitting during work, motorised transport or watching TV, is the largest contributor to inactivity [4,5]. Higher levels of SB have been associated with poor physical and mental health, increased risk of chronic disease and less successful ageing [6-9]. Consequently, several countries, including the UK, have issued recommendations to reduce SB at all ages as part of their national physical activity guidelines [10]. Population surveillance is urgently needed to monitor the impact of such policy, track changes in SB over time, and to evaluate public health interventions targeting SB. In order to provide effective surveillance upon which to base future policy decisions, such surveillance tools should be accurate (provide a true measure of the actual amount of SB in a population) and sensitive to change (provide the true difference in SB between two measurement time points) [11].

Objective body worn sensors, that measure posture, demonstrate good accuracy for measuring total duration of SB against the gold standard of direct observation [12], but they are expensive and challenging to use for population surveillance. Self-report tools provide a pragmatic choice for population surveillance and have the potential to provide context rich information, useful for intervention development [13]. To date, surveys assessing SB have predominantly used self-report tools [14], which are generally adapted from tools not specifically designed to measure that behaviour (e.g. tools designed to measure physical activity) [15], and which have not been evaluated for population surveillance purposes [14]. No framework currently exists with which to describe and compare SB self-report tools, meaning there is currently no way of systematically selecting an appropriate tool. A previous systematic review of the measurement characteristics of self-report tools measuring SB, reported acceptable to good reliability but low to moderate correlation with a

For peer review only - http://bmjopen.bmj.com/site/about/guidelines.xhtml

(non-gold standard) criterion measure [13]. This suggests that self-report measures of SB are acceptable tools to establish epidemiological evidence of an association between SB and health [13]. However, it is possible that the scale of the problem may be vastly underestimated, as differences of 2-4 hours per day (approximately 20% of SB) have been reported between self-report and objective tools [16].

The primary aim of this study was to identify, in a systematic manner, the optimal tool to measure SB for use in population surveillance. To fulfil the primary aim, a framework was created to describe the features of self-report tools measuring SB, the TAxonomy of Self-report Sedentary behaviour Tools (TASST). A systematic inventory of existing self-report tools to measure SB was mapped onto TASST, and the measurement characteristics of these tools, focussing on accuracy and sensitivity to change, were evaluated, with explicit reference to the domains of the taxonomy framework.

## METHODS

The study protocol (PROSPERO CRD42014009851), was conducted in three phases. In phase 1 an exhaustive inventory of self-reported tools to measure SB in adults and older adults was established using a structured search protocol. Phase 2 was the development of a taxonomy based on content analysis of the items and questions in the tools. In phase 3, a systematic literature review of the measurement characteristics of the tools in the inventory was conducted and mapped onto the taxonomy.

### Phase 1: Systematic inventory of self-report tools

For peer review only

112 The aim of the systematic inventory was to compile an exhaustive list of self-report tools which could be used to measure SB in adults and older adults. Since the aim was to identify tools and not to identify articles, this stage does not have the same methodology as a systematic literature review. A literature search was conducted in October 2013, for articles reporting SB as an outcome measure. From this review, an list of self-report tools which measured SB was compiled. References lists were reviewed and experts consulted to identify any additional tools to include in the inventory. The inventory then was consolidated to amalgamate tools referred to by different names, and to trace back to the original version. Articles which added significant new questions to tools were included as a separate tool. Tools used in a single study and those without names/acronyms were included as separate tools.

123 To be included in the inventory, tools had to: be suitable for use for large scale population studies of adults or older adults; measure SB or a proxy measure of SB; and be suitable for self-completion by the respondent at a single point in time. Tools were excluded from the inventory: if they were designed specifically to assess SB in children or other specialised populations (e.g. medical conditions); if they were designed specifically to assess SB in a specialised context, (e.g. workplace or care settings); if continuous reporting over extended periods of time was required (e.g. diaries or time-use surveys); or if significant interviewer interactions were required. Self-report tools that could be administered by telephone or interview were not automatically excluded, however tools such as the PDR (Previous Day Recall) [17], in which the interviewer works through lists of several hundred items, were excluded.

134 **Phase 2: Development of a taxonomy**

For peer review only - http://bmjopen.bmj.com/site/about/guidelines.xhtml

The original text was extracted for each question relating to SB in each of the self-report tools identified in the inventory. Content analysis was conducted on the text to extract all of the attributes in the questions that were used to describe and constrain what aspect of SB was measured by that question. For example, in the question "During the last 7 days, how much time did you usually spend sitting on a week day?", attributes extracted relating to the measurement of SB would be "during the last 7 days", "time spent sitting" and "on a week day". Attributes were then grouped into mutually exclusive domains covering similar aspects of measurement, and categories within those domains were defined iteratively. A new category was created each time a tool did not fit within an existing category. The full taxonomy was then assembled and streamlined by merging categories with overlapping meaning. Finally, consideration was given to potential future developments of self-report tools to measure SB, by adding any categories to the taxonomy considered useful in the future. The resulting taxonomy was then tested by ensuring that all tools could be classified similarly by two independent researchers and that the taxonomy fully defined the tool.

**Phase 3: Systematic review of measurement characteristics**

Finally, a systematic literature search in relevant health databases was conducted in December 2014 via EBSCO host. The search combined the name of the tool including variants and acronyms (except where the acronym was also a common word, e.g. PAST, MOST), with search terms relating to measurement characteristics (valid* /reliab* /repons* /sensitiv* /calibrat* /accura* /agreement /psychometric* /clinimetric* /"measurement characteristics" /Reliability and Validity (MeSH)). Articles were included only if they reported in English on the accuracy of a tools in the inventory against an objective criterion measure of SB, and/or sensitivity to change..

For peer review only

Exclusion by title, then abstract, then full-text was conducted independently by two researchers from a pool of four [PD, EC, CF, SC]. In the case of disagreement, the article was carried forward in to the next round, or at full-text stage a third researcher was consulted to ensure consensus. Data (tool, criterion, population, statistical analysis, accuracy of sedentary behaviour, sensitivity to change of sedentary behaviour) was extracted and quality was assessed independently by two researchers from a pool of three [PD, CF, SC]. Disagreements were resolved by discussion. Quality was assessed using QUALSYST [18], modified to include an additional item for the criterion measure. As per the QUALSYST guidelines, the quality score for the article (range 0-1) was used to identify common methodological strengths and flaws, rather than as an objective representation of high/low quality. Accuracy and sensitivity to change extracted from included articles were reported for tools in relation to the TASST taxonomy.

## RESULTS

### Inventory

The systematic inventory identified 32 distinct self-report tools used to measure SB in adults and older adults (Table 1). The International Physical Activity Questionnaire (IPAQ) has four different versions included in the inventory (combinations of the long and short versions, and last seven days and usual week recall). The 45 and Up study asked different questions in its baseline and follow-up questionnaires, which have been included as separate tools. Three tools, termed “modified” versions, were included where questions had been added or modified to the original tool (EPAQ2, IPAQ-L, NHANES), and were considered to form a substantially different version. Some tools identified were used in only a single study, and these were included in the inventory, referred to by the study name. These 32 tools comprised of 141 individual questions, consisting of between 1 and

For peer review only - http://bmjopen.bmj.com/site/about/guidelines.xhtml

20 questions per tool. An evaluation of the content of these individual items formed the basis of the TASST taxonomy.

For peer review only

For peer review only - http://bmjopen.bmj.com/site/about/guidelines.xhtml

Table 1 Tools measuring SB for population surveillance identified in the inventory

| Acronym | Name of Tool/Study | Key reference |
|---|---|---|
| 45Up-B | 45 and Up study, baseline questionnaire | [19] |
| 45Up-F | 45 and Up study, follow up questionnaire | [19] |
| ACS2 | American Cancer Society, Cancer Prevention Study cohort II | [20] |
| ALTS | Australian Leisure Time Sitting questionnaire | [21] |
| AusDiab | The Australian Diabetes Obesity and Lifestyle study | [22] |
| CCHS | Canadian Community Health Survey | [23] |
| CFS | Canadian Fitness Survey | [24] |
| CHAMPS | Community Health Activities Model Program for Seniors physical activity questionnaire | [15] |
| ELSA | English Longitudinal Study of Ageing | [25] |
| EPAQ2 | European Prospective Investigation of Cancer (EPIC)-Norfolk Physical Activity Questionnaire | [26] |
| mod EQPAQ2 | modified version of the EPIC-Norfolk Physical Activity Questionnaire | [27] |
| GPAQ | Global Physical Activity Questionnaire | [28] |
| HSE | Health Survey for England | [29] |
| HUNT3 | Nord-Trøndelag Health Study 3 | [30] |
| IPAQ-L l7d | International Physical Activity Questionnaire, Long version, last 7 days | [31] |
| IPAQ-L uw | International Physical Activity Questionnaire, Long version, usual week | [31] |
| mod IPAQ-L | modified version of the International Physical Activity Questionnaire, Long version | [32] |
| IPAQ-S l7d | International Physical Activity Questionnaire, Short version, last 7 days | [31] |
| IPAQ-S uw | International Physical Activity Questionnaire, Short version, usual week | [31] |
| LASA | Longitudinal Aging Study Amsterdam | [33] |
| MLTPAQ | Minnesota Leisure Time Physical Activity Questionnaire | [34] |
| MOST | Measuring Older adults' Sedentary Time questionnaire | [35] |
| NHANES | National Health and Nutrition Examination Survey | [36] |
| mod NHANES | modified version of the National Health and Nutrition Examination Survey | [37] |
| NHS2 | Nurses Health Survey II | [38] |
| NIH-AARP DHS | National Institutes of Health – American Association of Retired Persons (NIH-AARP) Diet and Health Survey | [39] |
| NSWPAS | New South Wales Physical Activity Survey | [40] |
| PASE | Physical Activity Scale for the Elderly | [41] |
| PAST | Past-day Adults Sedentary Time questionnaire | [42] |
| PCSpa | prospective cohort study (Spain) | [43] |
| SBQ | Sedentary Behaviour Questionnaire | [44] |
| SHS | Scottish Health Survey | [45] |

Acronym: the commonly used acronym of the tool, or the short identifier adopted for this article Name of Tool: either the name of the tool, or the name of the single study using these questions/tool. Key reference: references provided here are not exhaustive, but refer either to an early or well cited description of the tool, or the study in which the tool was used or adapted.

For peer review only - http://bmjopen.bmj.com/site/about/guidelines.xhtml

**TAxonomy for Self-report Sedentary behaviour Tools (TASST)**

The taxonomy derived from the inventory of self-report tools to measure SB (Figure 1) comprises of four domains, which characterise different aspects of the tool: type of assessment, recall period, temporal unit, and assessment period. All four aspects are required to describe the tool. Within each aspect, the taxonomy functions as a tree, meaning you can identify a single end point (taxon) which fully describes each question in a tool..

The type of assessment domain of the taxonomy covers the way that the outcome of time spent in SB is derived from the tool. Tools can either ask about a single aspect of SB (1.1 single item), or a composite aspect (1.2 composite). Tools using a single item of assessment will generate all of their information about SB within the relevant period of assessment in a single question. That single item can either ask about sitting time directly (1.1.1 direct measure) or it can ask about a single behaviour related to SB which is then used as a proxy measure of SB duration (1.1.2 proxy measure). Composite items of assessment ask multiple questions about several aspects of SB for the same period of assessment. One form of composite item would be to ask about the pattern (i.e. frequency and timing) of SB accumulated throughout the recall period (1.2.1 pattern). However, the most common form of composite item is created as a sum (1.2.2 sum) of the time spent in SB in a range of different activities or situations. The sum can either be formed from questions asking about specific behaviours (1.2.2.1), activities such as TV viewing, hobbies, talking with friends, or they can be based on domains (1.2.2.2), locations or situations where you can sit, such as at home, for transport and at work.

The recall period is total time over which the respondent is asked to consider their SB when answering the questions. The recall period can be anchored to the present time in which case it

For peer review only

refers to a specific length of time prior to now, for example yesterday (2.1 previous day), last week (2.2 previous week), or a longer period such as the last month or year (2.3 longer). The recall period can also be unanchored (2.4), in which case the respondent is not asked about a specific period but is asked about a general period of time, for example asking about SB in a typical week.

The temporal unit is the duration within the recall period that a respondent is asked to report their SB for. For example, in the question "on a typical day last week, how long did you sit?" the recall period is the previous week, but the temporal unit is a day. Within the taxonomy, the temporal units may be a day (3.1), a week (3.2) or longer (3.3). Within a particular recall period, it is possible to have any temporal unit that is of identical or shorter duration than the recall period.

The period of assessment is completed by identifying any specific restrictions that are placed on the type of temporal unit recalled. The categories within the assessment period domain clarify whether a respondent is asked questions regarding a particular type of day, for example only about week days (4.1), only weekend days (4.2), or is asked about weekdays and weekend days in separate questions (4.3 both). Additionally, the assessment period domain can identify if a respondent is asked about particular sub divisions of the day (4.4) in separate questions, for example time spent sitting before 6pm. The final taxon in the assessment period is termed 'not defined' (4.5), this represents the situation where a respondent is asked about all temporal units (e.g. days) within the recall period (e.g. last week) without any specific distinction being made between them. It is a global category, which usually represents a decision not to separate out these categories, as opposed to a failure to define this domain.

**Mapping the Inventory on to the Taxonomy**

For peer review only - http://bmjopen.bmj.com/site/about/guidelines.xhtml

The 32 tools identified in the inventory were mapped against the TASST taxonomy (Table 2). Over half of the tools in the inventory (n=17) used a single item of assessment, thirteen used a direct measure and seven used a proxy measure. Three tools asked single item questions about both a direct measure and a proxy measure, but not in a manner in which they could be used as a sum, and have therefore been included in the count for both taxa. Proxy measures were predominantly based on TV viewing (n=5). Fifteen tools used composite assessment, all of which used a sum as that composite item. The vast majority of sums were formed from questions asking about different behaviours (n=14), with only one sum formed from questions asking about different domains. The tools using a sum of behaviours generally included the common proxy measures of TV viewing (n=14) and computer use (n=12) within the sum. Many tools included questions for behaviours based on leisure pursuits (n=9), in social contexts (n=6), and during transportation (n=8). Often several behaviours of each type were considered in separate questions (e.g. asking about time sitting while reading separately from time spent sitting listening to music). Questions based on time working were included in five tools, but were explicitly excluded in four tools. Less frequently, tools included questions based on rest (n=2), or used an "other" category to cover circumstances not explicit within the questions (n=3).

For peer review only

Table 2: Mapping of the tools measuring SB identified in the inventory onto the TASST taxonomy.

| Taxonomy Item | | N | Tools | Accuracy | Sensitivity to change |
|---|---|---|---|---|---|
| **1** | **Type of Assessment** | | | | |
| 1.1 | Single item | 17 | | **Underestimate with large systematic and a random error** | + |
| 1.1.1 | Direct measure | 13 | 45Up-B; ACS2; AusDiab; CFS; GPAQ; HUNT3; IPAQ-L l7d; IPAQ-L uw; IPAQ-S l7d; IPAQ-S uw; NIH-AARP DHS; PASE; PCSPa | | |
| 1.1.2 | Proxy measure | 7 | 45Up-B; AusDiab; ELSA; MLTPAQ; NIH-AARP DHS; NSWPAS; SHS | | |
| 1.2 | Composite item | 15 | | **Smaller systematic error but there is a potential to overestimate** | + |
| 1.2.1 | Pattern | 0 | | | |
| 1.2.2 | Sum | 15 | | | |
| 1.2.2.1 | Behaviours | 14 | 45Up-F; ALTS; CCHS; CHAMPS; EPAQ2; mod EPAQ2; HSE; mod IPAQ-L; LASA; MOST; NHANES; mod NHANES; PAST; SBQ; | | |
| 1.2.2.2 | Domains | 1 | NHS2 | | |
| **2** | **Recall period** | | | | |
| 2.1 | Previous day | 1 | PAST | **+** | - |
| 2.2 | Previous week | 8 | 45Up-F; ALTS; AusDiab; IPAQ-L l7d; IPAQ-S l7d; mod IPAQ-L; MOST; PASE | **-** | + |
| 2.3 | Longer | 8 | ACS2; CCHS; CHAMPS; EPAQ2; mod EPAQ2; HSE; NHANES; NIH-AARP DHS | **-** | - |
| 2.4 | Unanchored | 15 | 45Up-B; CFS; ELSA; GPAQ; HUNT3; IPAQ-L uw; IPAQ-S uw; LASA; MLTPAQ; mod NHANES; NHS2; NSWPAS; PCSpa; SBQ; SHS | | |
| **3** | **Temporal Unit** | | | | |
| 3.1 | Day | 27 | 45Up-B; 45Up-F; ACS2; AusDiab; CFS; ELSA; EPAQ2; mod EPAQ2; GPAQ; HSE; HUNT3; IPAQ-L l7d; IPAQ-L uw; IPAQ-S l7d; IPAQ-S uw; mod IPAQ-L; LASA; MLTPAQ; NHANES; mod NHANES; NIH-AARP DHS; NSWPAS; PASE; PAST; PCSpa; SBQ; SHS | **+** | + |
| 3.2 | Week | 5 | ALTS; CCHS; CHAMPS; MOST; NHS2 | - | - |
| 3.3 | Longer | 0 | | - | - |
| **4** | **Assessment Period** | | | | |
| 4.1 | Weekdays only | 2 | IPAQ-S l7d; IPAQ-S uw | - | + |
| 4.2 | Weekend days only | 0 | | - | - |
| 4.3 | Both weekdays and weekend days | 12 | 45Up-F; AusDiab; ELSA; HSE; IPAQ-L l7d; IPAQ-L uw; mod IPAQ-L; LASA; NSWPAS; PCSpa; SBQ; SHS | + | - |
| 4.4 | Subdivision of the day | 1 | EPAQ2 | + | - |
| 4.5 | Not defined | 18 | 45Up-B; ACS2; ALTS; CCHS; CFS; CHAMPS; EPAQ2; mod EPAQ2; GPAQ; HUNT3; MLTPAQ; MOST; NHS2; NHANES; mod NHANES; NIH-AARP DHS; PASE; PAST | **Better for older adults** | + |

Full names for the acronyms reported in the Tools column can be found in Table 1. Recommendations in bold are backed by evidence from the systematic review. Recommendations which are not bold are theoretical but no evidence could be found in the literature. + represents a positive attribute; – a negative attribute.



For peer review only - http://bmjopen.bmj.com/site/about/guidelines.xhtml

About half the tools in the inventory used an unanchored recall period (n=15), eight used a previous week recall period, and eight used a longer recall period. Only a single tool in the inventory used a previous day recall period. The majority of tools used a temporal unit of a day (n=27), with five using a temporal unit of a week. A single question within the EPAQ2 questionnaire was based on a temporal unit longer than a week, but the other three questions in that tool were based on a temporal unit of a day. Just over half the tools (n=18) did not define specific days or time periods in their questions, but asked about the temporal unit within the recall period as a single entity. Fourteen tools used questions specifically referring to week or weekend days, twelve asking about both week and weekend days, while two asked only about week days. Only one tool referred to specific sub-divisions of the day in their questions.

**Systematic search for measurement characteristics**

The systematic search returned 5,640 references, and after removal of duplicate and assessment against exclusion criteria, a total of 14 studies were included in the review (figure 2, table 3).

For peer review only

Table 3: Measurement characteristics of tools measuring SB, presented by tool and taxon

| Tool | Taxon (refer to figure 1) | N | Population *(Country)* | Criterion measure (definition of SB) | QUALSYST Score | Agreement *(hours/day)* tool - criterion [limit of agreement] | Sensitivity to change | Ref |
|---|---|---|---|---|---|---|---|---|
| IPAQ-Long l7d | 1.1.1/2.2/3.1/4.3 | 1508 | A & OA (Greenland) | actiHeart (<1.5MET) | 0.67 | -3.0 [not reported] for adults<br>-6.0 [not reported] for older adults | -- | [46] |
| | | 542 | A (Netherlands) | Actigraph (<100 count/min) | 0.78 | -1.6 [-6.4 3.2] | -- | [47] |
| | | 980 | A (Sweden) | Actigraph (<100 count/min) | 0.67 | +2.2 [-4.5 9.5] | -- | [48] |
| | | 69 | A (UK) | activPAL (sitting/lying postures) | 0.78 | -2.2 [-7.22 3.71] | -- | [16] |
| | | 317 | A (Chile) | Actigraph (<100 count/min) | 0.78 | -1.1 [-3.8 1.5] | -- | [49] |
| IPAQ-Short l7d | 1.1.1/2.2/3.1/4.1 | 1751 | A & OA (Norway) | Actigraph (<100 count/min) | 0.67 | -1.8 [not reported] for adults<br>+3.5 [not reported] for older adults | -- | [50] |
| | | 144 | A (Nigeria) | Actigraph (<100 count/min) | 0.78 | -3.0 [-8.5 2.5] | -- | [51] |
| | | 54 | OA (Sweden) | Actigraph (<100 count/min) | 0.56 | -1.5 [not reported] | -- | [52] |
| | | 127 | OA (USA) | Actigraph (<50 count/min) | 0.72 | -4.4 [-10.0 -1.4] | -- | [53] |
| CHAMPS | 1.2.2.1/2.3/3.2/4.5 | 870 | OA (USA) | Actigraph (<100 count/min) | 0.72 | -6.8 [-10.6 2.4] | -- | [15] |
| | | 58 | OA (USA) | Actigraph (<100 count/min) | 0.72 | -5.2 [not reported] | -- | [54] |
| LASA | 1.2.2.1/2.4/3.1/4.3 | 83 | OA (Netherlands) | Actigraph (<100 count/min) | 0.78 | + 0.2 for 10 item<br>-2.1 [-7.4 3.25] for 6 item | -- | [33] |
| PAST | 1.2.2.1/2.1/3.1/4.3 | 90 | A (Australia) | activPAL (sitting/lying postures) | 0.72 | -1.0 [- 5.75 3.76] | t-test was inconclusive | [42] |
| MOST | 1.2.2.1/2.2/3.2/4.5 | 48 | OA (Australia) | Actigraph (<100 count/min) | 0.67 | -3.6 [-7.4 -0.2] | Guyatt Index 0.39 (0.47 for Actigraph) | [35] |

A: adults; N: number of participants; OA: older adults; Ref: reference; UK: United Kingdom; USA: United States of America. For tool acronyms see table 1.

For peer review only

## Criterion measure

None of the studies tested the accuracy of the tool against direct observation. Only two studies.[16,42]used a postural sensor that actually measures sitting time objectively (activPAL), the other twelve used an accelerometer built to measure low movement as a criterion measure (ActiGraph, actiheart).

## Statistical analysis

Accuracy and Limits of Agreement were usually derived from Bland and Altman plots. Sensitivity to change was defined differently in the two articles which reported this measurement characteristic; one used t-test statistics [42], one used the Guyatt Index [35].

## Study Quality

Studies which scored highly for quality tended to be purposefully designed to test measurement characteristics, rather than secondary analysis of data collected for another purpose. The most common loss of quality was due to the use of accelerometers which assess low movement (e.g. ActiGraph) as a criterion measure, as this does not measure the primary aspect of the definition of SB (i.e. posture). Another issue which lowered quality was the manipulation of the criterion measure without clear justification. For example, some studies manipulated the count threshold (used to define SB) or included only SB bouts longer than a particular duration without justification or solid rationale.

## Tools and measurement characteristics

For peer review only

Table 3 summarises the results reported by these studies, arranged per measurement tool and mapped against the relevant taxon. Very few of the existing tools to measure SB using self-report have actually been investigated for these measurement characteristics. Accuracy has been reported for six out of the 32 tools identified in the inventory (IPAQ-L l7d, IPAQ-S l7d, MOST, CHAMPS, LASA, PAST). The most tested tool was the IPAQ in its long form, last seven days [16, 4-9] and short form, last seven days.[5-3] CHAMPS was investigated in two studies [15, 53]. Information for other tools; LASA [33], MOST [35], PAST [42], come from single studies. Reports of sensitivity to change are only available for two tools; MOST [35] and PAST [42].

Taxa tested

The literature provides measurement characteristics information for six distinct full taxa:

1.1.1/2.2/3.1/4.3 with five studies on IPAQ-L uw;

1.1.1/2.2/3.1/4.1 with four studies on IPAQ-S uw

1.2.2.1/2.3/3.2/4.5 with two studies on CHAMPS;

1.2.2.1/2.4/3.1/4.3 with one study on LASA;

1.2.2.1/2.1/3.1/4.3 with one study on PAST; and

1.2.2.1/2.2/3.2/4.5 with one study on MOST.

For the assessment type, there is information for direct measures via single item (1.1.1, nine studies) and for composite sums of behaviours (1.2.2.1, five studies). However, there is no information for direct proxy measures (1.1.2). For recall period, there is information on all four possible categories (2.1 previous day, one study; 2.2 previous week, ten studies; 2.3 longer, three studies; and 2.4 unanchored, one study). The unanchored recall period (2.4), used by half of the tools in the

For peer review only - http://bmjopen.bmj.com/site/about/guidelines.xhtml

inventory, is particularly under-represented with only a single study in the validation review. For temporal scale there is mostly information for assessment at day scale (3.1, twelve studies) and only two studies for the temporal scale of a week (3.2). This is broadly representative of usage by tools in the inventory. For assessment period there is information for weekdays only (4.1, four studies) or both weekdays and weekend days (4.3, seven studies) and for tools with undefined assessment periods (4.5, three studies). The not defined taxon (4.5) is under-represented by these validation studies.

Accuracy

Information for taxon 1.1.1/2.2/3.1/4.3 is not equivocal. The majority of studies reported a large underestimation of total SB time ranging from 1.6 hours in adults [47] to 6 hours in older adults [46], Others report that tools in this taxon overestimate total SB time by 2.2 hours in adults [48]. While the direction of the error is equivocal it is clear that the systematic error on estimates of total SB time using tools from this taxon is likely to be very large (several hours/day). The random error is also likely to be very large as the Limits of Agreement reported were consistently very large. Information for taxon 1.1.1/2.2/3.1/4.1 is a little more consistent for adults. Tools in this taxon seem to underestimate total SB time by 1.5 to 3 hours in adults. However, in older adults this was less clear with reports of underestimation by 4.4 hours [53] and overestimation by 3.5 hours [50]. In both populations the error and Limits of Agreement were large, but not as large as for the previous taxon.

For taxon 1.2.2.1/2.3/3.2/4.5 there is consistent evidence for poor accuracy and large underestimation of 5 to 7 hours/day in older adults. This implies that a longer recall period or the temporal scale of a week might be less accurate. Other taxa under the 1.2.2.1 (sum of behaviours) categories report smaller errors ranging from 0.2 hours overestimate [33] to a 3.6 hour

For peer review only

underestimation [35], both in older adults. One study [42], which used an objective measure of sitting as criterion, reported a 1 hour underestimate and the smallest limit of agreement. This suggests that tools in taxa with the attributes of 1.2.2.1 (sum of behaviours) and 3.1 (assessment at day scale) are more likely to lead to accurate estimates of total SB time for a population.

Sensitivity to change

There is almost no information about sensitivity to change. The two studies that assessed sensitivity to change [35, 42] provided little tangible information. The results were either inconclusive [42], or reported the Guyatt index against a criterion measure which does not measure sitting [35]. While the latter provided some indication that the tools' sensitivity to change was similar to that of an objective measure of low movement it does not give a clear indication as to whether it is sensitive to a change in total SB time. Neither of these studies reported the minimal detectable change [55], a metric which provides an easily interpretable value of the capacity of a tool to detect a change.

## DISCUSSION

A taxonomy (TASST) for the systematic description and comparison of self-reported measures of SB has been established. TASST provides a rigorous framework for informed choice, development and evaluation of self-report tools. This framework has been used to review the measurement characteristics of existing tools in order to identify the optimum tool for population surveillance. The available evidence about measurement characteristics essential for population surveillance, namely accuracy and responsiveness to change, was insufficient to ascertain which tool currently used in practice is best.  Accuracy was poor for all existing tools, with both under and over estimation of total time spent in SB and large limits of agreement. In addition, there is a complete lack of evidence about their sensitivity to change. Mapping available evidence onto the TASST framework has

For peer review only - http://bmjopen.bmj.com/site/about/guidelines.xhtml

enabled informed recommendations to be made about the promising features for a surveillance tool, and identification of the aspects on which future research and development of SB surveillance tools should focus.

The use of a coherent and robust taxonomy (TASST) to systematically evaluate and compare the characteristics of measurement tools is the main strength of this study. However, in terms of accuracy and sensitivity to change, the current published evidence does not cover the entire taxonomy. Consequently, at present, only tentative recommendations can be provided. The taxonomy can be used, however, to identify gaps in current research and provide focussed guidance for future research and development. During the development of TASST, self-report tools which aimed to measure SB in specific populations (e.g. children, those with arthritis) or specialised contexts (e.g. workplace) were not considered. However, TASST is a generic framework, so tools specific to these populations may already be fully described by the taxonomy. For example, a question asking about time spent sitting at school which is specific to children, would be covered under the sub-division of the day assessment period (taxon 4.4). Another consequence of the exclusion criteria is that evidence on accuracy and sensitivity to change of tools specific to these populations was not mapped on the taxonomy. Therefore, the conclusions drawn from the measurement characteristics in this study are only valid for adults and older adults. In addition, this study has the general limitations common to most systematic reviews, i.e. included articles were restricted to those written in English, articles and tools published after the date of search were not included, and any relevant articles not identified during the search will have been excluded.

The current study is the first to clearly define and focus on the measurement characteristics required for population surveillance (accuracy and sensitivity to change). There is only one other systematic

For peer review only

review reporting on the measurement characteristics of self-report tools to measure SB [13], which concentrated on validity (assessed through rank correlation) and reliability, which are the measurement characteristics relevant to establishing association between SB and health. In agreement with the previous review, we found that the major flaw of most validation studies was the use of an inadequate criterion measure. The choice of criterion measure depends on the purpose of the tool. While direct observation should be considered the gold standard, if the purpose is to assess total sedentary time, then accurate postural sensors should be adequate (e.g. activPAL). Instead, many studies used an accelerometer which measures low levels of movement at the hip (e.g. ActiGraph) as a criterion measure, but such tools do not measure SB directly and can misclassify standing as sitting [12].

Despite the incomplete nature of the evidence, TASST enables the identification of desirable characteristics of self-report tools to measure SB when used for population surveillance. Firstly, tools assessing total SB time as a sum of behaviours (taxon 1.2.2.1) provided better accuracy than single item (taxon 1.1) tools. However, this will be dependent on the behaviours or domains included within the sum, and whether they are exhaustive, consistent and mutually exclusive. Tools with a non-exhaustive sum will underestimate total time, for example, the Longitudinal Aging Study Amsterdam (LASA), found that a six item sum provided a better correlation with SB across the sample, but that a ten item sum was more accurate [33]. Conversely, tools which contain behaviours which might occur concurrently (such as watching TV and using a tablet computer) may lead to an over-estimate in total SB time [56]. Secondly, tools using a previous day recall period (taxon 2.1) provide better accuracy than those with longer recall periods (taxa 2.2 and 2.3). This corroborates recent research on the validity of computerised survey systems which assess SB using a past-day recall period [17, 57]. However, although tools using previous day recall may more

For peer review only

accurate, it is likely that their sensitivity to change will be less good due to the higher underlying variability in daily SB [58].

Most tools currently used for population surveillance of SB systematically underestimate the amount of SB by two to four hours per day. Yet, self-report tools are still the most practical and economical means of population surveillance. Therefore, policy makers and clinicians should be aware that reports of population SB time are likely to be grossly underestimated, and should be cognisant of this fact when making decisions on implementing, developing and evaluating policy and public health interventions. In addition, policy makers and clinicians should be cautious in interpreting any reported difference in population SB time as a real change. The dearth of information about sensitivity to change of these tools means that we do not know the magnitude of change required to be certain that a change is real and not background variation. Moving forward, development of national and international surveillance systems should not be undertaken assuming that a tool is adequate because it has been used previously. Instead, investment should be made in research to evaluate the sensitivity to change and accuracy of tools to measure SB, paying attention to the potential trade-off between these two measurement characteristics. Such research should be carefully planned, to ensure that meaningful comparisons are investigated. The TASST taxonomy should be used as a useful framework to facilitate such a systematic approach.

For peer review only

432 Acknowledgements

433 The named authors present the study on behalf of the Seniors USP Team, which comprises: Dawn A Skelton (PI), Sebastien Chastin, Simon Cox, Elaine Coulter, Iva Cukic, Philippa Dall, Ian Deary, Geoff Der, Manon Dontje, Claire Fitzsimons, Catherine Gale, Jason Gill, Malcolm Granat, Cindy Gray, Carolyn Greig, Elaine Hindle, Karen Laird, Gillian Mead, Nanette Mutrie, Victoria Palmer, Ratko Radakovic, Naveed Sattar, Richard Shaw, John Starr, Sally Stewart, Sally Wyke.

439 Competing Interests:

440 All authors had financial support from the Medical Research Council for the submitted work; no financial relationships with any organisations that might have an interest in the submitted work in the previous three years; no other relationships or activities that could appear to have influenced the submitted work.

445 Funding:

446 The study was performed in preparation for the Seniors USP: Understanding Sedentary Patterns project, which was supported by the Medical Research Council [MR/K025023/1]

449 Authors Contributions:

450 SC and DS conceived and supervised the study. PD and SC designed the study. PD, EC, CF and SC collected the data, and performed the systematic review. PD, CF and SC analysed the data for the taxonomy. PD and SC analysed the data from the systematic review. All authors interpreted the data and critically revised the manuscript for important intellectual content.

For peer review only

Data Sharing: Data can be obtained from the corresponding author on request.

Figure Legends

Figure 1: TAxonomy of Self-reported Sedentary behaviour Tools (TASST)

Figure 2: PRISMA diagram of the validation systematic review

For peer review only

463 References

464

465 1. Kohl HW 3rd, Craig CL, Lambert EV, et al. The pandemic of physical inactivity: global action for
466 public health. *Lancet* 2012;380:294–305.

467 2. Chastin S, Scwartz U, Skelton D. Development of a consensus taxonomy of sedentary behaviors
468 (SIT): report of Delphi round 1. *PLoS One* 2013;8:e82313.

469 3. Sedentary Behaviour Research Network. Standardized use of the terms "sedentary" and
470 "sedentary behaviours". *Appl Physiol Nutr Metabol* 2012;37:540-2.

471 4. Healy GN, Matthews CE, Dunstan DW, Winkler EAH, Owen N. Sedentary time and cardio-
472 metabolic biomarkers in US adults: NHANES 2003-06. *Eur Heart J* 2011;32:590-7.

473 5. Harvey JA, Chastin SFM, Skelton DA. How sedentary are older people? A systematic review of the
474 amount of sedentary behavior. *J Aging Phys Act* 2015;23:471-87.

475 6. Proper KI, Singh AS, van Mechelen W, Chinapaw MJM. Sedentary behaviors and health outcomes
476 among adults: a systematic review of prospective studies. *Am J Prev Med* 2011,40:174-82.

477 7. Thorp AA, Owen N, Neuhaus M, Dunstan DW. Sedentary behaviors and subsequent health
478 outcomes in adults a systematic review of longitudinal studies, 1996-2011. *Am J Prev Med*
479 2011;41:207-15.

480 8. de Rezende LFM, Rey-López JP, Matsudo VKR, do Carmo Luiz O. Sedentary behavior and health
481 outcomes among older adults: a systematic review. *BMC Public Health* 2014;14:333.

482 9. Dogra S, Stathokostas L. Sedentary behavior and physical activity are independent predictors of
483 successful aging in middle-aged and older adults. *J Aging Res* 2012;2012:190654.

For peer review only

484 10. Department of Health. Start Active, Stay Active: A report on physical activity from the four home
485 countries' Chief Medical Officers. 2011.

486 11. Guyatt G, Walter S, Norman G. Measuring change over time. assessing the usefulness of
487 evaluative instruments. *J Chron Dis* 1987;40:171-8.

488 12. Kozey-Keadle S, Libertine A, Lyden K, Staudenmayer J, Freedson P. Validation of wearable
489 monitors for assessing sedentary behavior. *Med Sci Sports Exerc* 2011;43:1561-7.

490 13. Healy GN, Clark BK, Winkler EAH, Gardiner PA, Brown WJ, Matthews CE. Measurement of adults'
491 sedentary time in population-based studies. *Am J Prev Med* 2011,;41:216-27.

492 14. Bauman A, Ainsworth BE, Sallis JF, et al. The descriptive epidemiology of sitting: a 20 country
493 comparison using the International Physical Activity Questionnaire (IPAQ). *Am J Prev Med*
494 2011;41:228-35.

495 15. Hekler EB, Buman MP, Haskell WL, et al. Reliability and validity of CHAMPS self-reported
496 sedentary-vigorous intensity physical activity in older adults. *J Phys Act Health* 2012;9:225-36.

497 16. Chastin SFM, Culhane B, Dall PM. Questionnaire sitting time (IPAQ) compared to objective
498 measurement via inclinometry (activPAL). *Physiol Meas* 2014;35:2319-28.

499 17. Kozey-Keadle S, Lyden K, Hickey A, et al. Validation of a previous day recall for measuring the
500 location and purpose of active and sedentary behaviours compared to direct observation. *Int J*
501 *Behav Nutr Phys Act* 2014;11:12.

502 18. Kmet LM, Lee RC, Cook LS. Standard quality assessment criteria for evaluating primary research
503 papers from a variety of fields. *Edmonton:* Alberta Heritage Foundation for Medical Research
504 2004;1-22.

505 19. Banks E, Jorm L, Rogers K, Clements M, Bauman A. Screen-time, obesity, ageing and disability:
506 findings from 91 266 participants in the 45 and Up Study. *Public Health Nutr* 2011;14:34-43.

For peer review only

507 20 Patel AV, Bernstein L, Deka A, et al. Leisure time spent sitting in relation to total mortality in a
508 prospective cohort of US adults. *Am J Epidemiol* 2010;172:419-429.

509 21. Salmon J, Owen N, Crawford D, Bauman A, Sallis JF. Physical activity and sedentary behavior: a
510 population-based study of barriers, enjoyment, and preference. *Health Psychol* 2003;22:178-88.

511 22. Gardiner PA, Healy GN, Eakin EG, et al. Associations between television viewing time and overall
512 sitting time with the metabolic syndrome in older men and women: The Australian Diabetes Obesity
513 and Lifestyle study. *JAGS* 2011;59:788-96.

514 23. Shields M, Tremblay MS. Sedentary behaviour and obesity. *Health Rep.* 2008;19:19-30.

515 24. Katzmarzyk PT, Church TS, Craig CI, Bouchard C. Sitting time and mortality from all causes,
516 cardiovascular disease, and cancer. *Med Sci Sports Exerc* 2009;41:998-1005.

517 25. Hamer M, Stamatakis E. Screen-based sedentary behaviour, physical activity, and muscle
518 strength in the English Longitudinal Study of Ageing. *PLoS One* 2013;8:e66222.

519 26. Wareham NJ, Jakes RW, Rennie KL, Mitchell J, Hennings S, Day NE. Validity and repeatability of
520 the EPIC-Norfolk physical activity questionnaire. *Int J Epidemiol* 2002;31:186-74.

521 27. Parsons TJ, Thomas C, Power C. Estimated activity patterns in British 45 year olds: cross-
522 sectional findings from the 1958 British birth cohort. *Eur J Clin Nutr* 2009;63:978-85.

523 28. Armstrong T, Bull F. Development of the World Health Organization Global Physical Activity
524 Questionnaire (GPAQ). *J Public Health* 2006;14:66-70.

525 29. Stamatakis E, Davis M, Stathi A, Hamer M. Associations between multiple indicators of
526 objectively-measured and self-reported sedentary behaviour and cardiometabolic risk in older
527 adults. *Prev Med* 2012;54:82-7.

For peer review only

30. Chau JY, Grunseit A, Midthjell K, et al. Sedentary behaviour and risk of mortality from all-causes and cardiometabolic diseases in adults: evidence from the HUNT3 population cohort. *Br J Sports Med* 2015;49:737-42.

31. Craig CL, Marshall AL, Sjöström M, et al. International Physical Activity Questionnaire: 12-country reliability and validity. *Med Sci Sports Exerc* 2003;35:1381-95.

32. Proper KI, Cerin E, Brown WJ, Owen N. Sitting time and socio-economic differences in overweight and obesity. *Int J Obes* 2007;31:169-76.

33. Visser M, Koster A. Development of a questionnaire to assess sedentary time in older persons – a comparative study using accelerometry. *BMC Geriatr* 2013;13:80.

34. Burazeri G, Goda A, Kark JD. Television viewing, leisure-time exercise and acute coronary syndrome in transitional Albania. *Prev Med* 2008;47:112-5.

35. Gardiner PA, Clark BK, Healy GN, Eakin EG, Winkler EAH, Owen N. Measuring older adults' sedentary time: reliability, validity, and responsiveness. *Med Sci Sports Exerc* 2011;43: 2127-33.

36. Ford ES. Combined television viewing and computer use and mortality from all-causes and diseases of the circulatory system among adults in the United States. *BMC Public Health* 2012;12:70

37. Evenson KR, McGinn AP. Test-retest reliability of adult surveillance measures for physical activity and inactivity. *Am J Prev Med* 2005;28:470-8.

38. Wolf AM, Hunter DJ, Colditz GA, et al. Reproducibility and validity of a self-administered physical activity questionnaire. *Int J Epidemiol* 1994;23:991-99.

39. Matthews CE, George SM, Moore SC, et al. Amount of time spent in sedentary behaviors and cause-specific mortality in US adults. *Am J Clin Nutr* 2012;ajcn-019620.


For peer review only

40. Salmon J, Bauman A, Crawford D, Timperio A, Owen N. The association between television viewing and overweight among Australian adults participating in varying levels of leisure-time physical activity. *Int J Obes Relat Metab Disord* 2000;24:600-6.

41. Washburn RA, McAuley E, Katula J, Mihalko SL, Boileau RA. The Physical Activity Scale for the Elderly (PASE): evidence for validity. *J Clin Epidemiol* 1999;52:643-51.

42. Clark BK, Winkler E, Healy GN, et al. Adults' past-day recall of sedentary time: reliability, validity and responsiveness. *Med Sci Sports Exerc* 2013;45:1198-207.

43. Balboa-Castillo T, León-Muñoz LM, Graciani A, Rodríguez-Artalejo F, Guallar-Castillón P. Longitudinal association of physical activity and sedentary behavior during leisure time with health-related quality of life in community-dwelling older adults. *Health Qual Life Outcomes* 2011;9:41-47.

44. Rosenberg DE, Norman GK, Wagner N, Patrick K, Calfas KJ, Sallis JF. Reliability and validity of the Sedentary Behaviour Questionnaire (SBQ) for adults. *J Phys Act Health* 2010;7:697-705.

45. Stamatakis E, Hamer M, Dunstan DW. Screen-based entertainment time, all-cause mortality, and cardiovascular events. *J Am Coll Cardiol* 2011;57:292-9.

46. Dahl-Pederson KI, Hansen AW, Bjerregaard P, Jørgensen ME, Brage S. Validity of the international physical activity questionnaire in the arctic. *Med Sci Sports Exerc* 2013;45:728-36.

47. van Dyck D, Cardon G, Deforche B, de Bourdeaudhuij I. IPAQ interview version: convergent validity with accelerometers and comparison of physical activity and sedentary time levels with the self-administered version. *J Sports Med Phys Fitness* 2014;55:776-86.

48. Hagstromer M, Ainsworth BE, Sjostrom M. Comparison of a subjective and an objective measure of physical activity in a population sample. *J Phys Act Health* 2010;7:541-50.

For peer review only

49. Celis-Morales CA, Perez-Bravo F, Ibanez L, Salas C, Bailey MES, Gill JMR. Objective vs. self-reported physical activity and sedentary time: effects of measurement method on relationships with risk biomarkers. *PLoS ONE* 2012;7:e36345.

50. Dyrstad SM, Hansen BH, Holme IM, Anderssen SA. Comparison of self-reported versus accelerometer-measured physical activity. *Med Sci Sports Exerc* 2014;46:99-106.

51. Oyeyemi AL, Umar M, Oguche F, Aliyu SU, Oyeyemi AY. Accelerometer-determined physical activity and its comparison with the International Physical Activity Questionnaire in a sample of Nigerian adults. *PLoS ONE* 2014;9:e87233.

52. Hurtig-Wennlöf A, Hagströmer M, Olsson L. The International Physical Activity Questionnaire modified for the elderly: aspects of validity and feasibility. *Public Health Nutr* 2010;13:1847-54.

53. Grimm EK, Swartz AM, Hart T, Miller NE, Strath SJ. Comparison of the IPAQ-short form and accelerometry predictions of physical activity in older adults. *J Aging Phys Act* 2012;20:64-79.

54. Gennuso KP, Matthews CE, Colbert LH. Reliability and validity of two self-report measures to assess sedentary behavior in older adults. *J Phys Act Health* 2015;12:727-32.

55. Beaton DE, Bombardier C, Katz JN, Wright JG. A taxonomy for responsiveness. *J Clin Epidemiol* 2001;54:1204-71.

56. Busschaert C, de Bourdeaudhuij I, van Holle V, Chastin SFM, Cardon G, de Cocker K. Reliability and validity of three questionnaires measuring context-specific sedentary behaviour and associated correlates in adolescents, adults, and older adults. *Int J Behav Nutr Phys Act* 2015;12:117.

57. Gomersall SR, Pavey T, Clark BK, Jasman A, Brown WJ. Validity of a self-report recall tool for estimating sedentary behaviour in adults. *J Phys Act Health* 2015;12:1485-91.

58. Hart TL, Swartz AM, Cashin SE, Strath SJ. How many days of monitoring predict physical activity and sedentary behaviour in older adults? *Int J Behav Nutr Phys Act* 2011;8:62.

For peer review only

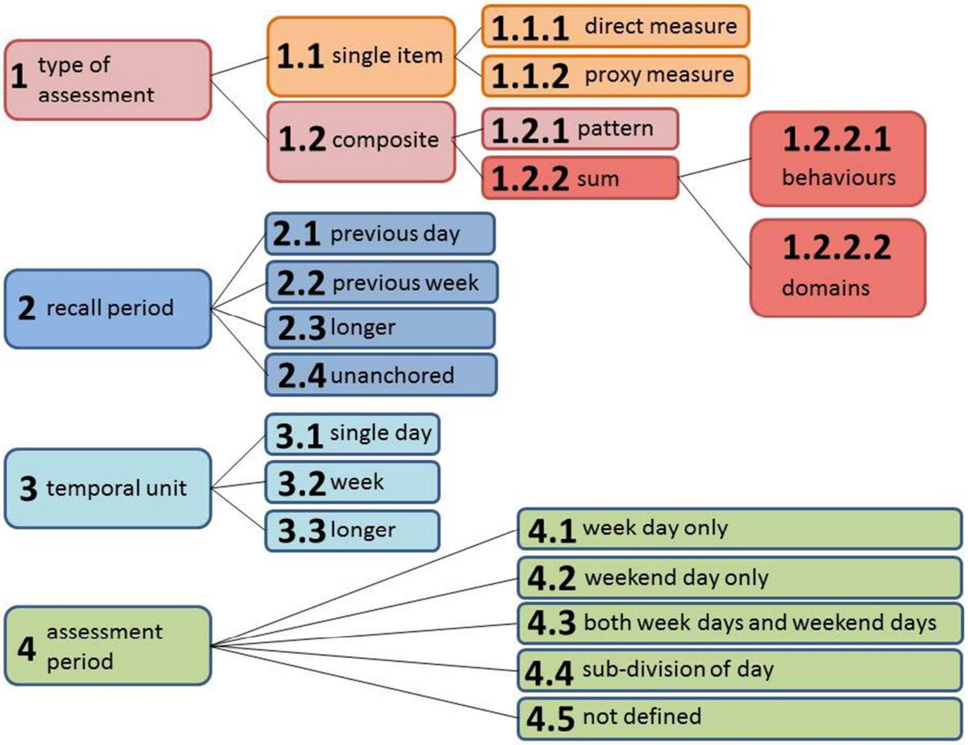


TAxonomy of Self-reported Sedentary behaviour Tools (TASST)
Figure 1
254x190mm (96 x 96 DPI)

For peer review only - http://bmjopen.bmj.com/site/about/guidelines.xhtml

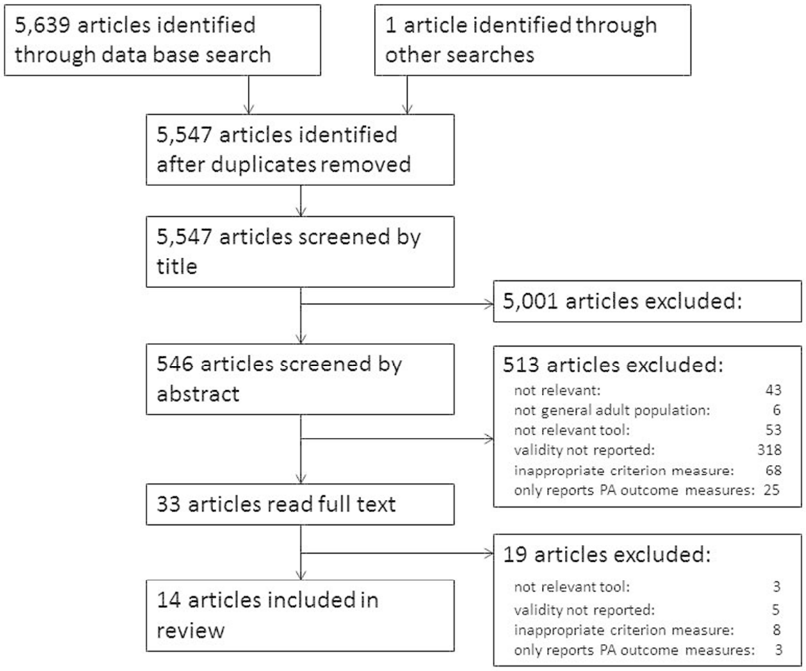


PRISMA diagram of the validation systematic review
Figure 2
254x190mm (96 x 96 DPI)

For peer review only - http://bmjopen.bmj.com/site/about/guidelines.xhtml

# PRISMA 2009 Checklist

| Section/topic | # | Checklist item | Reported on page # |
|---|---|---|---|
| **TITLE** | | | |
| Title | 1 | Identify the report as a systematic review, meta-analysis, or both. | 1 |
| **ABSTRACT** | | | |
| Structured summary | 2 | Provide a structured summary including, as applicable: background; objectives; data sources; study eligibility criteria, participants, and interventions; study appraisal and synthesis methods; results; limitations; conclusions and implications of key findings; systematic review registration number. | 2 |
| **INTRODUCTION** | | | |
| Rationale | 3 | Describe the rationale for the review in the context of what is already known. | 3 |
| Objectives | 4 | Provide an explicit statement of questions being addressed with reference to participants, interventions, comparisons, outcomes, and study design (PICOS). | 3 |
| **METHODS** | | | |
| Protocol and registration | 5 | Indicate if a review protocol exists, if and where it can be accessed (e.g., Web address), and, if available, provide registration information including registration number. | 4 |
| Eligibility criteria | 6 | Specify study characteristics (e.g., PICOS, length of follow-up) and report characteristics (e.g., years considered, language, publication status) used as criteria for eligibility, giving rationale. | 4 & 5 |
| Information sources | 7 | Describe all information sources (e.g., databases with dates of coverage, contact with study authors to identify additional studies) in the search and date last searched. | 4 & 5 |
| Search | 8 | Present full electronic search strategy for at least one database, including any limits used, such that it could be repeated. | 5 |
| Study selection | 9 | State the process for selecting studies (i.e., screening, eligibility, included in systematic review, and, if applicable, included in the meta-analysis). | 5 |
| Data collection process | 10 | Describe method of data extraction from reports (e.g., piloted forms, independently, in duplicate) and any processes for obtaining and confirming data from investigators. | 5 |
| Data items | 11 | List and define all variables for which data were sought (e.g., PICOS, funding sources) and any assumptions and simplifications made. | 5 |
| Risk of bias in individual studies | 12 | Describe methods used for assessing risk of bias of individual studies (including specification of whether this was done at the study or outcome level), and how this information is to be used in any data synthesis. | Not reporting on trials, so not done |

For peer review only

# PRISMA 2009 Checklist

| | | | |
|---|---|---|---|
| Summary measures | 13 | State the principal summary measures (e.g., risk ratio, difference in means). | 5 |
| Synthesis of results | 14 | Describe the methods of handling data and combining results of studies, if done, including measures of consistency (e.g., $I^2$) for each meta-analysis. | Not done |



| Section/topic | # | Checklist item | Reported on page # |
|---|---|---|---|
| Risk of bias across studies | 15 | Specify any assessment of risk of bias that may affect the cumulative evidence (e.g., publication bias, selective reporting within studies). | 9 |
| Additional analyses | 16 | Describe methods of additional analyses (e.g., sensitivity or subgroup analyses, meta-regression), if done, indicating which were pre-specified. | Not done |
| **RESULTS** | | | |
| Study selection | 17 | Give numbers of studies screened, assessed for eligibility, and included in the review, with reasons for exclusions at each stage, ideally with a flow diagram. | 8, figure 2 |
| Study characteristics | 18 | For each study, present characteristics for which data were extracted (e.g., study size, PICOS, follow-up period) and provide the citations. | Table 3 |
| Risk of bias within studies | 19 | Present data on risk of bias of each study and, if available, any outcome level assessment (see item 12). | 8 |
| Results of individual studies | 20 | For all outcomes considered (benefits or harms), present, for each study: (a) simple summary data for each intervention group (b) effect estimates and confidence intervals, ideally with a forest plot. | Table 3, pages 9 & 10 (not a review of interventions) |
| Synthesis of results | 21 | Present results of each meta-analysis done, including confidence intervals and measures of consistency. | Not enough data for meta-analysis |
| Risk of bias across studies | 22 | Present results of any assessment of risk of bias across studies (see Item 15). | 9 |
| Additional analysis | 23 | Give results of additional analyses, if done (e.g., sensitivity or subgroup analyses, meta-regression [see Item 16]). | Not done |
| **DISCUSSION** | | | |
| Summary of evidence | 24 | Summarize the main findings including the strength of evidence for each main outcome; consider their relevance to key groups (e.g., healthcare providers, users, and policy makers). | 12 |
| Limitations | 25 | Discuss limitations at study and outcome level (e.g., risk of bias), and at review-level (e.g., incomplete retrieval of identified research, reporting bias). | 11 & 12 |

For peer review only

# PRISMA 2009 Checklist

| | | | |
|---|---|---|---|
| Conclusions | 26 | Provide a general interpretation of the results in the context of other evidence, and implications for future research. | 12 |
| **FUNDING** | | | |
| Funding | 27 | Describe sources of funding for the systematic review and other support (e.g., supply of data); role of funders for the systematic review. | 14 |

*From:* Moher D, Liberati A, Tetzlaff J, Altman DG, The PRISMA Group (2009). Preferred Reporting Items for Systematic Reviews and Meta-Analyses: The PRISMA Statement. PLoS Med 6(6): e1000097. doi:10.1371/journal.pmed1000097

For more information, visit: www.prisma-statement.org.

Page 2 of 2

For peer review only

BMJ Open

# TAxonomy of Self-reported Sedentary behaviour Tools (TASST): a framework for development, comparison and evaluation

| | |
|---|---|
| Journal: | *BMJ Open* |
| Manuscript ID | bmjopen-2016-013844.R1 |
| Article Type: | Research |
| Date Submitted by the Author: | 30-Nov-2016 |
| Complete List of Authors: | Dall, Philippa; Glasgow Caledonian University School of Health and Life Sciences, Department of Psychology, Social Work & Allied Health Sciences<br>Coulter, Elaine; University of Glasgow<br>Fitzsimons, Claire F.; Univ Edinburgh, ISPEHS<br>Skelton, Dawn; Glasgow Caledonian University, School of Health & Life Sciences<br>Chastin, Sebastian; Glasgow Caledonian University |
| <b>Primary Subject Heading</b>: | Public health |
| Secondary Subject Heading: | Epidemiology, Research methods, Sports and exercise medicine |
| Keywords: | sedentary behaviour, sitting, population surveillance, measurement, validation |
| | |

SCHOLARONE™
Manuscripts

For peer review only

**Title:** TAxonomy of Self-reported Sedentary behaviour Tools (TASST): a framework for development, comparison and evaluation

**Authors:**

Dall PM[1], Coulter EH[2], Fitzsimons CF[3], Skelton DA[1], Chastin SFM[1], on behalf of the Seniors USP Team.

[1] Institute for Applied Health Research, Glasgow Caledonian University, Cowcaddens Road, Glasgow, G4 0BA

[2] Department of Nursing and Healthcare, Glasgow University, 59 Oakfield Avenue, Glasgow, G12 8LL

[3] Sport, Physical Education and Health Sciences Institute, Edinburgh University, St Leonard's Land, Holyrood Road, Edinburgh, EH8 8AQ

corresponding author:

Dr Philippa Dall

School of Health & Life Sciences

Glasgow Caledonian University

Cowcaddens Road

Glasgow

G4 0BA

philippa.dall@gcu.ac.uk

Word count: 5,035

For peer review only

**ABSTRACT**

**Objective:** Sedentary behaviour (SB) has distinct deleterious health outcomes, yet there is no consensus on best practice for measurement. This study aimed to identify the optimal self-report tool for population surveillance of SB, using a systematic framework.

**Design:** A framework, TAxonomy of Self-report SB Tools (TASST), consisting of four domains (type of assessment, recall period, temporal unit, and assessment period), was developed based on a systematic inventory of existing tools. The inventory was achieved through a systematic review of studies reporting SB and tracing back to the original description. A systematic review of the accuracy and sensitivity to change of these tools was then mapped against TASST domains.

**Data Sources:** Systematic searches were conducted via EBSCO, reference lists and expert opinion.

**Eligibility Criteria for selecting studies:** The inventory included tools measuring SB in adults that could be self-completed at one sitting, and excluded tools measuring SB in specific populations or contexts. The systematic review included studies reporting on the accuracy against an objective measure of SB and/or sensitivity to change of a tool in the inventory.

**Results:** The systematic review initially identified 32 distinct tools (141 questions), which were used to develop the TASST framework. Twenty-two studies evaluated accuracy and/or sensitivity to change representing only 8 taxa. Assessing SB as a sum of behaviours and using a previous day recall were the most promising features of existing tools. Accuracy was poor for all existing tools, with both under and over estimation of SB. There was a lack of evidence about sensitivity to change.

**Conclusions:** Despite the limited evidence, mapping existing SB tools onto the TASST framework has enabled informed recommendations to be made about the most promising features for a surveillance tool, identified aspects on which future research and development of SB surveillance tools should focus.

For peer review only

**Systematic Review Registration**

PROSPERO/CRD42014009851

**KEY WORDS:**

sedentary behaviour; sitting; population surveillance; measurement; validation

**STRENGTHS AND LIMITATIONS OF THIS STUDY:**

- A systematic approach was taken towards classifying self-reported measures of sedentary behaviour, allowing a structured approach to measurement in the future
- An example of use of the framework is presented, mapping accuracy and sensitivity to change of self-report sedentary behaviour measures on to the framework
- Although designed to be generic, the TASST framework was developed excluding tools measuring sedentary behaviour in specialised populations and contexts, e.g. children or the workplace, and the framework may therefore not cover some aspects of these tools
- There is the potential for a language bias, as full-text articles not in English were not included in the systematic reviews.

For peer review only - http://bmjopen.bmj.com/site/about/guidelines.xhtml

## BACKGROUND

Physical inactivity is currently at pandemic levels [1] and is a global public health concern. Sedentary behaviour (SB), an umbrella term for all waking time spent in non-exercising sitting or reclining postures [2, 3] such as sitting during work, motorised transport or watching TV, is the largest contributor to inactivity [4,5]. Higher levels of SB have been associated with poor physical and mental health, increased risk of chronic disease and less successful ageing [6-9]. Consequently, several countries, including the UK, have issued recommendations to reduce SB at all ages as part of their national physical activity guidelines [10]. Population surveillance is urgently needed to monitor the impact of such policy, track changes in SB over time, and to evaluate public health interventions targeting SB. In order to provide effective surveillance upon which to base future policy decisions, such surveillance tools should be accurate (provide a true measure of the actual amount of SB in a population) and sensitive to change (provide the true difference in SB between two measurement time points) [11].

Objective body worn sensors, that measure posture, demonstrate good accuracy for measuring total duration of SB against the gold standard of direct observation [12], but they are expensive and challenging to use for population surveillance. Self-report tools provide a pragmatic choice for population surveillance and have the potential to provide context rich information, useful for intervention development [13]. To date, surveys assessing SB have predominantly used self-report tools [14], which are generally adapted from tools not specifically designed to measure that behaviour (e.g. tools designed to measure physical activity) [15], and which have not been evaluated for population surveillance purposes [14]. No framework currently exists with which to describe and compare SB self-report tools, meaning there is currently no way of systematically selecting an appropriate tool. A previous systematic review of the measurement characteristics of self-report tools measuring SB, reported acceptable to good reliability but low to moderate correlation with a

For peer review only

(non-gold standard) criterion measure [13]. This suggests that self-report measures of SB are acceptable tools to establish epidemiological evidence of an association between SB and health [13]. However, it is possible that the scale of the problem may be vastly underestimated, as differences of 2-4 hours per day (approximately 20% of SB) have been reported between self-report and objective tools [16].

The primary aim of this study was to identify, in a systematic manner, the optimal self-report tool to measure SB for use in population surveillance. Although self-report SB tools can and will be used in other areas of research, this study focussed on population surveillance as an area that is crucial to the development of public health policy. To fulfil the primary aim, a framework was created to describe the features of self-report tools measuring SB, the TAxonomy of Self-report Sedentary behaviour Tools (TASST). A systematic inventory of existing self-report tools to measure SB was mapped onto TASST, and the measurement characteristics of these tools, focussing on accuracy and sensitivity to change, were evaluated, with explicit reference to the domains of the taxonomy framework.

**METHODS**

The study protocol (PROSPERO CRD42014009851), was conducted in three phases. In phase 1 an exhaustive inventory of self-reported tools to measure SB in adults and older adults was established using a structured search protocol. Phase 2 was the development of a taxonomy based on content analysis of the items and questions in the tools. In phase 3, a systematic literature review of the measurement characteristics of the tools in the inventory was conducted and mapped onto the taxonomy.

For peer review only

**Phase 1: Systematic inventory of self-report tools**

The aim of the systematic inventory was to compile an exhaustive list of self-report tools which could be used to measure SB in adults (≥18 years) and older adults(≥60 years). Since the aim was to identify tools and not to identify articles, this stage does not have the same methodology as a systematic literature review. A literature search was conducted in October 2013 (updated November 2016), for articles reporting SB as an outcome measure. From this review, a list of self-report tools which measured SB was compiled. References lists were reviewed and experts consulted to identify any additional tools to include in the inventory. The inventory then was consolidated to amalgamate tools referred to by different names, and to trace back to the original version. Articles which added significant new questions to tools were included as a separate tool. We defined significant new questions to be at least one question which added or changed the type of sedentary behaviour or the time period considered by the tool. Changes in phrasing of the question were not considered sufficient to be considered as a separate tool. Tools used in a single study and those without names/acronyms were included as separate tools.

To be included in the inventory, tools had to: be suitable for use for large scale population studies of adults or older adults, including being suitable for self-completion by the respondent at a single point in time (a pragmatic requirement to minimise participant burden); and measure SB or a proxy measure of SB (e.g. TV viewing). Tools were excluded from the inventory: if they were designed specifically to assess SB in children or other specialised populations (e.g. medical conditions); if they were designed specifically to assess SB in a specialised context, (e.g. workplace or care settings); if continuous reporting over extended periods of time was required (e.g. diaries or time-use surveys); or if significant interviewer interactions were required. Self-report tools that could be administered by telephone or interview were not automatically excluded, however tools such as the PDR (Previous

For peer review only - http://bmjopen.bmj.com/site/about/guidelines.xhtml

Day Recall) [17], in which the interviewer works through lists of several hundred items, were excluded.

**Phase 2: Development of a taxonomy**

Only tools identified in the initial search were used to develop the taxonomy. The original text was extracted for each question relating to SB in each of the self-report tools identified in the inventory. Content analysis was conducted on the text to extract all of the attributes in the questions that were used to describe and constrain what aspect of SB was measured by that question. For example, in the question "During the last 7 days, how much time did you usually spend sitting on a week day?", attributes extracted relating to the measurement of SB would be "during the last 7 days", "time spent sitting" and "on a week day". Attributes were then grouped into mutually exclusive domains covering similar aspects of measurement, and categories within those domains were defined iteratively. A new category was created each time a tool did not fit within an existing category. The full taxonomy was then assembled and streamlined by merging categories with overlapping meaning. Finally, consideration was given to potential future developments of self-report tools to measure SB, such as the growing interest in the pattern of accumulation of sedentary behaviour, by adding any categories to the taxonomy considered useful in the future. The resulting taxonomy was then tested by ensuring that all tools could be classified similarly by two independent researchers and that the taxonomy fully defined the tool.

**Phase 3: Systematic review of measurement characteristics**

Finally, a systematic literature search in relevant health databases was conducted in December 2014 (updated November 2016) via EBSCO host. The search combined the name of the tool including variants and acronyms (except where the acronym was also a common word, e.g. PAST, MOST), with

For peer review only

search terms relating to measurement characteristics (valid* /reliab* /repons* /sensitiv* /calibrat* /accura* /agreement /psychometric* /clinimetric* /"measurement characteristics" /Reliability and Validity (MeSH)). Articles were included only if they reported in English on the accuracy of a tools in the inventory against an objective criterion measure of SB, and/or sensitivity to change. Although articles were only included in the review if they assessed accuracy or sensitivity to change, the search terms included a wide range of psychometric properties in order to maximise the chances of finding eligible articles.

Exclusion by title, then abstract, then full-text was conducted by two researchers from a pool of five [PD, EC, CF, SC, CL]. In the case of disagreement, the article was carried forward in to the next round, or at full-text stage a third researcher was consulted to ensure consensus. Data (tool, criterion, population, statistical analysis, accuracy of sedentary behaviour, sensitivity to change of sedentary behaviour) was extracted and quality was assessed independently by two researchers from a pool of three [PD, CF, SC]. Disagreements were resolved by discussion. Quality was assessed using QUALSYST [18], modified to include an additional item for the criterion measure. As per the QUALSYST guidelines, the quality score for the article (range 0-1) was used to identify common methodological strengths and flaws, rather than as an objective representation of high/low quality. Accuracy and sensitivity to change extracted from included articles were reported for tools in relation to the TASST taxonomy.

## RESULTS

### Inventory

The systematic inventory identified 37 distinct self-report tools used to measure SB in adults and older adults, 32 of which were identified in the initial search and used to form the taxonomy (Table

For peer review only

1). The International Physical Activity Questionnaire (IPAQ) was originally developed with four different versions, which were included separately in the inventory (combinations of the long and short versions, and last seven days and usual week recall). The 45 and Up study asked different questions in its baseline and follow-up questionnaires, which have been included as separate tools. Three tools, termed "modified" versions, were included where questions had been added or modified to the original tool (EPAQ2, NHANES, and IPAQ-L, representing a 5th version of the IPAQ in the inventory), and were considered to form a substantially different version. Some tools identified were used in only a single study, and these were included in the inventory, referred to by the study name. The 32 tools in the original inventory comprised of 141 individual questions, consisting of between 1 and 20 questions per tool. An evaluation of the content of these individual items formed the basis of the TASST taxonomy.

For peer review only

Table 1 Tools measuring SB for population surveillance identified in the inventory

| Acronym | Name of Tool/Study | Key reference |
|---|---|---|
| 45Up-B | 45 and Up study, baseline questionnaire | [19] |
| 45Up-F | 45 and Up study, follow up questionnaire | [19] |
| ACS2 | American Cancer Society, Cancer Prevention Study cohort II | [20] |
| ALTS | Australian Leisure Time Sitting questionnaire | [21] |
| AusDiab | The Australian Diabetes Obesity and Lifestyle study | [22] |
| CCHS | Canadian Community Health Survey | [23] |
| CFS | Canadian Fitness Survey | [24] |
| CHAMPS | Community Health Activities Model Program for Seniors physical activity questionnaire | [15] |
| ELSA | English Longitudinal Study of Ageing | [25] |
| EPAQ2 | European Prospective Investigation of Cancer (EPIC)-Norfolk Physical Activity Questionnaire | [26] |
| mod EQPAQ2 | modified version of the EPIC-Norfolk Physical Activity Questionnaire | [27] |
| GPAQ | Global Physical Activity Questionnaire | [28] |
| HSE | Health Survey for England | [29] |
| HUNT3 | Nord-Trøndelag Health Study 3 | [30] |
| IPAQ-L l7d | International Physical Activity Questionnaire, Long version, last 7 days | [31] |
| IPAQ-L uw | International Physical Activity Questionnaire, Long version, usual week | [31] |
| mod IPAQ-L | modified version of the International Physical Activity Questionnaire, Long version | [32] |
| IPAQ-S l7d | International Physical Activity Questionnaire,  Short version, last 7 days | [31] |
| IPAQ-S uw | International Physical Activity Questionnaire, Short version, usual week | [31] |
| LASA | Longitudinal Aging Study Amsterdam | [33] |
| MLTPAQ | Minnesota Leisure Time Physical Activity Questionnaire | [34] |
| MOST | Measuring Older adults' Sedentary Time questionnaire | [35] |
| NHANES | National Health and Nutrition Examination Survey | [36] |
| mod NHANES | modified version of the National Health and Nutrition Examination Survey | [37] |
| NHS2 | Nurses Health Survey II | [38] |
| NIH-AARP DHS | National Institutes of Health – American Association of Retired Persons (NIH-AARP) Diet and Health Survey | [39] |
| NSWPAS | New South Wales Physical Activity Survey | [40] |
| PASE | Physical Activity Scale for the Elderly | [41] |
| PAST | Past-day Adults Sedentary Time questionnaire | [42] |
| PAST-U* | Past-day Adults Sedentary Time questionnaire – University version | [43] |
| PCSpa | prospective cohort study (Spain) | [44] |
| SBQ | Sedentary Behaviour Questionnaire | [45] |
| SHS | Scottish Health Survey | [46] |
| SIT-Q* | SIT-Q | [47] |
| SIT-Q-7d* | past seven day version of the SIT-Q | [48] |
| STAR-Q* | Sedentary Time and Reporting Questionnaire | [49] |
| STAQ* | Sedentary, Transportation and Activity Questionnaire | [50] |

For peer review only - http://bmjopen.bmj.com/site/about/guidelines.xhtml

Acronym: the commonly used acronym of the tool, or the short identifier adopted for this article Name of Tool: either the name of the tool, or the name of the single study using these questions/tool. Key reference: references provided here are not exhaustive, but refer either to an early or well cited description of the tool, or the study in which the tool was used or adapted. Tools marked with an asterisk (*) were identified in the updated search, and were not used to create the taxonomy

For peer review only

**TAxonomy for Self-report Sedentary behaviour Tools (TASST)**

The taxonomy derived from the inventory of self-report tools to measure SB (Figure 1) comprises of four domains, which characterise different aspects of the tool: type of assessment, recall period, temporal unit, and assessment period. All four aspects are required to describe the tool. Within each aspect, the taxonomy functions as a tree, meaning you can identify a single end point (taxon) which fully describes each question in a tool.

The type of assessment domain of the taxonomy covers the way that the outcome of time spent in SB is derived from the tool. Tools can either ask about a single aspect of SB (1.1 single item), or a composite aspect (1.2 composite). Tools using a single item of assessment will generate all of their information about SB within the relevant period of assessment in a single question. That single item can either ask about sitting time directly (1.1.1 direct measure) or it can ask about a single behaviour related to SB which is then used as a proxy measure of SB duration (1.1.2 proxy measure). Composite items of assessment ask multiple questions about several aspects of SB for the same period of assessment. One form of composite item would be to ask about the pattern (i.e. frequency and timing) of SB accumulated throughout the recall period (1.2.1 pattern). However, the most common form of composite item is created as a sum (1.2.2 sum) of the time spent in SB in a range of different activities or situations. The sum can either be formed from questions asking about specific behaviours (1.2.2.1), activities such as TV viewing, hobbies, talking with friends, or they can be based on domains (1.2.2.2), locations or situations where you can sit, such as at home, for transport and at work.

The recall period is total time over which the respondent is asked to consider their SB when answering the questions. The recall period can be anchored to the present time in which case it

For peer review only - http://bmjopen.bmj.com/site/about/guidelines.xhtml

refers to a specific length of time prior to now, for example yesterday (2.1 previous day), last week (2.2 previous week), or a longer period such as the last month or year (2.3 longer). The recall period can also be unanchored (2.4), in which case the respondent is not asked about a specific period but is asked about a general period of time, for example asking about SB in a typical week.

The temporal unit is the duration within the recall period that a respondent is asked to report their SB for. For example, in the question "on a typical day last week, how long did you sit?" the recall period is the previous week, but the temporal unit is a day. Within the taxonomy, the temporal units may be a day (3.1), a week (3.2) or longer (3.3). Within a particular recall period, it is possible to have any temporal unit that is of identical or shorter duration than the recall period.

The period of assessment is completed by identifying any specific restrictions that are placed on the type of temporal unit recalled. The categories within the assessment period domain clarify whether a respondent is asked questions regarding a particular type of day, for example only about week days (4.1), only weekend days (4.2), or is asked about weekdays and weekend days in separate questions (4.3 both). Additionally, the assessment period domain can identify if a respondent is asked about particular sub divisions of the day (4.4) in separate questions, for example time spent sitting before 6pm. The final taxon in the assessment period is termed 'not defined' (4.5), this represents the situation where a respondent is asked about all temporal units (e.g. days) within the recall period (e.g. last week) without any specific distinction being made between them. It is a global category, which usually represents a decision not to separate out these categories, as opposed to a failure to define this domain.

**Mapping the Inventory on to the Taxonomy**

For peer review only

The 37 tools identified in the inventory were mapped against the TASST taxonomy (Table 2). Approximately half of the tools in the inventory (n=17) used a single item of assessment, thirteen used a direct measure and seven used a proxy measure. Three tools (45Up-B, AusDiab, NIH-AARP DHS) asked single item questions about both a direct measure and a proxy measure, but not in a manner in which they could be used as a sum, and have therefore been included in the count for both taxa. Proxy measures were predominantly based on TV viewing (n=5). Twenty tools used composite assessment, all of which used a sum as that composite item. The vast majority of sums were formed from questions asking about different behaviours (n=19), with only one sum formed from questions asking about different domains. The tools using a sum of behaviours generally included the common proxy measures of TV viewing (n=19) and computer use (n=17) within the sum. Many tools included questions for behaviours based on leisure pursuits (n=14), in social contexts (n=9), and during transportation (n=13). Often several behaviours of each type were considered in separate questions (e.g. asking about time sitting while reading separately from time spent sitting listening to music). Questions based on time working were included in ten tools, but were explicitly excluded in four tools. Less frequently, tools included questions based on rest (n=5), or used an “other” category to cover circumstances not explicit within the questions (n=7).

For peer review only

Table 2: Mapping of the tools measuring SB identified in the inventory onto the TASST taxonomy.

| Taxonomy Item | | N | Tools | Accuracy | Sensitivity to change |
|---|---|---|---|---|---|
| **1** | **Type of Assessment** | | | | |
| 1.1 | Single item | 17 | | **Underestimate with large systematic and a random error** | + |
| 1.1.1 | Direct measure | 13 | 45Up-B; ACS2; AusDiab; CFS; GPAQ; HUNT3; IPAQ-L l7d; IPAQ-L uw; IPAQ-S l7d; IPAQ-S uw; NIH-AARP DHS; PASE; PCSPa | | |
| 1.1.2 | Proxy measure | 7 | 45Up-B; AusDiab; ELSA; MLTPAQ; NIH-AARP DHS; NSWPAS; SHS | | |
| 1.2 | Composite item | 20 | | **Smaller systematic error but there is a potential to overestimate** | + |
| 1.2.1 | Pattern | 0 | | | |
| 1.2.2 | Sum | | | | |
| 1.2.2.1 | Behaviours | 19 | 45Up-F; ALTS; CCHS; CHAMPS; EPAQ2; mod EPAQ2; HSE; mod IPAQ-L; LASA; MOST; NHANES; mod NHANES; PAST; PAST-U; SBQ; SIT-Q; SIT-Q-7d; STAR-Q; STAQ | | |
| 1.2.2.2 | Domains | 1 | NHS2 | | |
| **2** | **Recall period** | | | | |
| 2.1 | Previous day | 2 | PAST; PAST-U | + | - |
| 2.2 | Previous week | 9 | 45Up-F; ALTS; AusDiab; IPAQ-L l7d; IPAQ-S l7d; mod IPAQ-L; MOST; PASE; SIT-Q-7d | - | + |
| 2.3 | Longer | 11 | ACS2; CCHS; CHAMPS; EPAQ2; mod EPAQ2; HSE; NHANES; NIH-AARP DHS; SIT-Q; STAR-Q; STAQ | - | - |
| 2.4 | Unanchored | 15 | 45Up-B; CFS; ELSA; GPAQ; HUNT3; IPAQ-L uw; IPAQ-S uw; LASA; MLTPAQ; mod NHANES; NHS2; NSWPAS; PCSpa; SBQ; SHS | | |
| **3** | **Temporal Unit** | | | | |
| 3.1 | Day | 32 | 45Up-B; 45Up-F; ACS2; AusDiab; CFS; ELSA; EPAQ2; mod EPAQ2; GPAQ; HSE; HUNT3; IPAQ-L l7d; IPAQ-L uw; IPAQ-S l7d; IPAQ-S uw; mod IPAQ-L; LASA; MLTPAQ; NHANES; mod NHANES; NIH-AARP DHS; NSWPAS; PASE; PAST; PAST-U; PCSpa; SBQ; SHS; SIT-Q; SIT-Q-7d; STAR-Q; STAQ | + | + |
| 3.2 | Week | 5 | ALTS; CCHS; CHAMPS; MOST; NHS2 | - | - |
| 3.3 | Longer | 0 | | - | - |
| **4** | **Assessment Period** | | | | |
| 4.1 | Weekdays only | 2 | IPAQ-S l7d; IPAQ-S uw | - | + |
| 4.2 | Weekend days only | 0 | | - | - |
| 4.3 | Both weekdays and weekend days | 14 | 45Up-F; AusDiab; ELSA; HSE; IPAQ-L l7d; IPAQ-L uw; mod IPAQ-L; LASA; NSWPAS; PCSpa; SBQ; SHS; SIT-Q-7d; STAQ | + | - |
| 4.4 | Subdivision of the day | 1 | EPAQ2 | + | - |
| 4.5 | Not defined | 21 | 45Up-B; ACS2; ALTS; CCHS; CFS; CHAMPS; EPAQ2; mod EPAQ2; GPAQ; HUNT3; MLTPAQ; MOST; NHS2; NHANES; mod NHANES; NIH-AARP DHS; PASE; PAST; PAST-U; SIT-Q; STAR-Q | **Better for older adults** | + |

Full names for the acronyms reported in the Tools column can be found in Table 1. Recommendations in bold are backed by evidence from the systematic review. Recommendations which are not bold are theoretical but no evidence could be found in the literature. + represents a positive attribute; – a negative attribute.



For peer review only - http://bmjopen.bmj.com/site/about/guidelines.xhtml

A little under half of the tools in the inventory used an unanchored recall period (n=15), nine used a previous week recall period, and eleven used a longer recall period. Only two tools (PAST, PAST-U) in the inventory used a previous day recall period. The majority of tools used a temporal unit of a day (n=32), with five (ALTS, CCHS, CHAMPS, MOST, NHS2) using a temporal unit of a week. A single question within the EPAQ2 questionnaire was based on a temporal unit longer than a week, but the other three questions in that tool were based on a temporal unit of a day. Just over half the tools (n=21) did not define specific days or time periods in their questions, but asked about the temporal unit within the recall period as a single entity. Sixteen tools used questions specifically referring to week or weekend days, fourteen asking about both week and weekend days, while two asked only about week days. Only one tool (EPAQ2) referred to specific sub-divisions of the day in their questions.

**Systematic search for measurement characteristics**

The systematic search returned 7,221 references, and after removal of duplicate and assessment against exclusion criteria (>99% agreement between reviewers), a total of 22 studies were included in the review (figure 2, table 3).

For peer review only

Table 3: Measurement characteristics of tools measuring SB, presented by tool and taxon

| Tool | Taxon (refer to figure 1) | N | Population *(Country)* | Criterion measure (definition of SB) | QUALSYST Score | Agreement *(hours/day)* tool - criterion [limit of agreement] | Sensitivity to change | Ref |
|---|---|---|---|---|---|---|---|---|
| IPAQ-Long l7d | 1.1.1/2.2/3.1/4.3 | 1508 | A & OA (Greenland) | actiHeart (<1.5MET) | 0.67 | -3.0 [not reported] for adults<br>-6.0 [not reported] for older adults | -- | [51] |
| | | 542 | A (Netherlands) | Actigraph (<100 count/min) | 0.78 | -1.6 [-6.4 3.2] | -- | [52] |
| | | 980 | A (Sweden) | Actigraph (<100 count/min) | 0.67 | +2.2 [-4.5 9.5] | -- | [53] |
| | | 69 | A (UK) | activPAL (sitting/lying postures) | 0.78 | -2.2 [-7.2 3.7] | -- | [16] |
| | | 317 | A (Chile) | Actigraph (<100 count/min) | 0.78 | -1.1 [-3.8 1.5] | -- | [54] |
| | | 346 | A & OA (Switzerland) | ActiGraph (<150 count/min) | 0.78 | -3.8 [-9.3 1.7] | -- | [55] |
| IPAQ-Short l7d | 1.1.1/2.2/3.1/4.1 | 1751 | A & OA (Norway) | Actigraph (<100 count/min) | 0.67 | -1.8 [not reported] for adults<br>+3.5 [not reported] for older adults | -- | [56] |
| | | 144 | A (Nigeria) | Actigraph (<100 count/min) | 0.78 | -3.0 [-8.5 2.5] | -- | [57] |
| | | 54 | OA (Sweden) | Actigraph (<100 count/min) | 0.56 | -1.5 [not reported] | -- | [58] |
| | | 127 | OA (USA) | Actigraph (<50 count/min) | 0.72 | -4.4 [-10.0 -1.4] | -- | [59] |
| | | 50 | A & OA (UK) | Actigraph (<50 count/min) | 0.72 | -0.5 [-1.9 0.8] | -- | [60] |
| GPAQ | 1.1.1/2.4/3.1/4.5 | 62 | A (Saudi Arabia) | Actigraph (<100 count/min) | 0.67 | -3.3 [-9.7 3.1] | -- | [61] |
| CHAMPS | 1.2.2.1/2.3/3.2/4.5 | 870 | OA (USA) | Actigraph (<100 count/min) | 0.72 | -6.8 [-10.6 2.4] | -- | [15] |
| | | 58 | OA (USA) | Actigraph (<100 count/min) | 0.72 | -5.2 [not reported] | -- | [62] |



For peer review only - http://bmjopen.bmj.com/site/about/guidelines.xhtml

| | | | | | | | | |
|---|---|---|---|---|---|---|---|---|
| LASA | 1.2.2.1/2.4/3.1/4.3 | 83 | OA (Netherlands) | Actigraph (<100 count/min) | 0.78 | + 0.2 for 10 item<br>-2.1 [-7.4 3.3] for 6 item | -- | [33] |
| STAQ | | 88 | A (France) | Actigraph (<150 count/min) | 0.72 | -2.4 [-6.2 4.9] | -- | [50] |
| PAST | 1.2.2.1/2.1/3.1/4.5 | 90 | A (Australia) | activPAL (sitting/lying postures) | 0.72 | -1.0 [- 5.6 3.8] | t-test was inconclusive | [42] |
| PAST-U | | 57 | A (Australia) | activPAL (sitting/lying postures) | 0.78 | 0.1 [-3.9 4.1] | -- | [43] |
| SIT-Q-7d | 1.2.2.1/2.2/3.1/4.3 | 51<br><br>402 | A (Belgium)<br>A (UK) | activPAL (sitting/lying postures)<br>actiHeart (<1.5MET) | 0.72 | 1.0 [-4.8 8.2] for Belgian sample<br><br>0.4 [-6.9 8.6] for UK sample | -- | [48] |
| | | 33 & 33 | A & OA (Belgium) | activPAL (sitting/lying postures) | 0.83 | 2.3 [only reported as a %]<br>0.3 [-8.9 0.7] for older adults | -- | [63] |
| | | 442 | OA (Belgium) | Actigraph (<100 count/min) | 0.83 | 1.36 [-6.0 3.3] | -- | [64] |
| MOST | 1.2.2.1/2.2/3.2/4.5 | 48 | OA (Australia) | Actigraph (<100 count/min) | 0.67 | -3.6 [-7.4 -0.2] | Guyatt Index 0.39 (0.47 for Actigraph) | [35] |

A: adults; N: number of participants; OA: older adults; Ref: reference; UK: United Kingdom; USA: United States of America. For tool acronyms see table 1.

For peer review only

Criterion measure

None of the studies tested the accuracy of the tool against direct observation. Only five studies [16,42,43,48,63] used a postural sensor that actually measures sitting time objectively (activPAL), the other seventeen used an accelerometer built to measure low movement as a criterion measure (ActiGraph, actiHeart).

Statistical analysis

Accuracy and Limits of Agreement were usually derived from Bland and Altman plots. Sensitivity to change was defined differently in the two articles which reported this measurement characteristic; one used t-test statistics [42], one used the Guyatt Index [35].

Study Quality

Studies which scored highly for quality tended to be purposefully designed to test measurement characteristics, rather than secondary analysis of data collected for another purpose. The most common loss of quality was due to the use of accelerometers which assess low movement (e.g. ActiGraph) as a criterion measure, as this does not measure the primary aspect of the definition of SB (i.e. posture). Another issue which lowered quality was the manipulation of the criterion measure without clear justification. For example, some studies manipulated the count threshold (used to define SB) or included only SB bouts longer than a particular duration without justification or solid rationale.

Tools and measurement characteristics

For peer review only

For peer review only - http://bmjopen.bmj.com/site/about/guidelines.xhtml

Table 3 summarises the results reported by these studies, arranged per measurement tool and mapped against the relevant taxon. Very few of the existing tools to measure SB using self-report have actually been investigated for these measurement characteristics. Accuracy has been reported for ten out of the 37 tools identified in the inventory (IPAQ-L l7d, IPAQ-S l7d, GPAQ, MOST, CHAMPS, LASA, PAST, PAST-U, STAQ, SIT-Q-7d). The most tested tools were the IPAQ in its long form, last seven days [16, 51-55] and short form, last seven days.[56-60]. The SIT-Q-7d was tested in three studies [48, 63-64], and the CHAMPS was investigated in two studies [15, 62]. Information for other tools; GPAQ [61], LASA [33], MOST [35], PAST [42], PAST-U [43], and STAQ [50], come from single studies. Reports of sensitivity to change are only available for two tools; MOST [35] and PAST [42].

Taxa tested

The literature provides measurement characteristics information for eight distinct full taxa:

- 1.1.1/2.2/3.1/4.3 with six studies on IPAQ-L uw;
- 1.1.1/2.2/3.1/4.1 with five studies on IPAQ-S uw;
- 1.1.1/2.4/3.1/4.5 with one study on GPAQ;
- 1.2.2.1/2.1/3.1/4.5 with one study on PAST and one study on PAST-U;
- 1.2.2.1/2.2/3.1/4.3 with three studies on SIT-Q-7d;
- 1.2.2.1/2.2/3.2/4.5 with one study on MOST;
- 1.2.2.1/2.3/3.2/4.5 with two studies on CHAMPS; and
- 1.2.2.1/2.4/3.1/4.3 with one study on LASA and one study on STAQ.

For peer review only

.

For the assessment type, there is information for direct measures via single item (1.1.1, twelve studies) and for composite sums of behaviours (1.2.2.1, ten studies). However, there is no information for direct proxy measures (1.1.2). For recall period, there is information on all four possible categories (2.1 previous day, two studies; 2.2 previous week, sixteen studies; 2.3 longer, two studies; and 2.4 unanchored, two studies). The unanchored recall period (2.4), used by 40% of the tools in the inventory, is particularly under-represented with only two studies in the validation review. For temporal scale there is mostly information for assessment at day scale (3.1, twenty studies) and only three studies for the temporal scale of a week (3.2). This is broadly representative of usage by tools in the inventory. For assessment period there is information for weekdays only (4.1, five studies) or both weekdays and weekend days (4.3, eleven studies) and for tools with the assessment period not defined (4.5, six studies). The assessment period not defined taxon (4.5), used by over half the tools in the inventory, is under-represented by these validation studies.

Accuracy

Information for taxon 1.1.1/2.2/3.1/4.3 (IPAQ-L-l7d) is not equivocal. The majority of studies reported a large underestimation of total SB time ranging from 1.1 hours in adults [54] to 6 hours in older adults [51]. One study reported that tools in this taxon overestimate total SB time by 2.2 hours in adults [53]. It is clear that the systematic error on estimates of total SB time using tools from this taxon is likely to be very large (several hours/day). The random error is also likely to be very large as the Limits of Agreement reported were consistently very large. Information for taxon 1.1.1/2.2/3.1/4.1 (IPAQ-S-l7d) is a little more consistent for adults. Tools in this taxon seem to underestimate total SB time by 1.5 to 3 hours in adults. However, in older adults this was less clear

For peer review only

with reports of underestimation by 4.4 hours [59] and overestimation by 3.5 hours [56]. In both populations the error and Limits of Agreement were large, but not as large as for the previous taxon.

Although not entirely consistent, tools reporting information from a single item as a direct measure of sitting (taxon 1.1.1) tended to underestimate sitting, with underestimation ranging from -0.5 [60] to -6.0 [51] hours per day. Within those tools, the IPAQ-S-l7d (reporting only for week days in the past week, taxa 2.2 & 4.1), tended to have better agreement than the IPAQ-L-17d (reporting for both week and weekend days in the past week, taxa 2.2 & 4.3), and the GPAQ (reporting over a longer recall period with the assessment period not defined, taxa 2.4 & 4.5). Tools reporting on a sum of behaviours (taxon 1.2.2.1), were more likely to overestimate sitting than for the single item direct measure (taxon 1.1.1). Tools which reported on a sum of behaviours over the past day or past week (taxa 1.2.2.1 & 2.1 or 2.2), tended to have the closest agreement with objective criterion measures, with most studies reporting agreement between -1.0 and +2.3 hours per day. Tools which reported sum of behaviours over a longer (taxon 2.3) or unanchored (taxon 2.4) recall period or which had a temporal unit of a week (taxon 3.2) reported larger underestimates (-2.1 to -6.8 hours/day). In particular the CHAMPS tool, reporting both for a recall period of a year (taxon 2.3) with a temporal unit of a week (taxon 3.2), had the largest differences for any tool. However, there were only a few studies reporting on these aspects, and such conclusions are necessarily tentative. Regardless of level of agreement, limits of agreement were large for all tools.

Sensitivity to change

There is almost no information about sensitivity to change. The two studies that assessed sensitivity to change [35, 42] provided little tangible information. The results were either inconclusive [42], or reported the Guyatt index against a criterion measure which does not measure sitting [35]. While

For peer review only - http://bmjopen.bmj.com/site/about/guidelines.xhtml

the latter provided some indication that the tools' sensitivity to change was similar to that of an objective measure of low movement it does not give a clear indication as to whether it is sensitive to a change in total SB time. Neither of these studies reported the minimal detectable change [65], a metric which provides an easily interpretable value of the capacity of a tool to detect a change.

## DISCUSSION

A taxonomy (TASST) for the systematic description and comparison of self-reported measures of SB has been established. TASST provides a rigorous framework for informed choice, development and evaluation of self-report tools. This framework has been used to review the measurement characteristics of existing tools in order to identify the optimum tool for population surveillance. The available evidence about measurement characteristics essential for population surveillance, namely accuracy and responsiveness to change, was insufficient to ascertain which tool currently used in practice is best. Accuracy was poor for all existing tools, with both under and over estimation of total time spent in SB and large limits of agreement. In addition, there is a complete lack of evidence about their sensitivity to change. Mapping available evidence onto the TASST framework has enabled informed recommendations to be made about the promising features for a surveillance tool, and identification of the aspects on which future research and development of SB surveillance tools should focus.

The use of a coherent and robust taxonomy (TASST) to systematically evaluate and compare the characteristics of measurement tools is the main strength of this study. However, in terms of accuracy and sensitivity to change, the current published evidence does not cover the entire taxonomy. Consequently, at present, only tentative recommendations can be provided. The taxonomy can be used, however, to identify gaps in current research and provide focussed guidance

For peer review only - http://bmjopen.bmj.com/site/about/guidelines.xhtml

for future research and development. During the development of TASST, self-report tools which aimed to measure SB in specific populations (e.g. children, those with arthritis) or specialised contexts (e.g. workplace) were not considered. However, TASST is a generic framework, so tools specific to these populations may already be fully described by the taxonomy. For example, a question asking about time spent sitting at school which is specific to children, would be covered under the sub-division of the day assessment period (taxon 4.4). Another consequence of the exclusion criteria is that evidence on accuracy and sensitivity to change of tools specific to these populations was not mapped on the taxonomy. Therefore, the conclusions drawn from the measurement characteristics in this study are only valid for adults and older adults. In addition, this study has the general limitations common to most systematic reviews, i.e. included articles were restricted to those written in English, articles and tools published after the date of search were not included, and any relevant articles not identified during the search will have been excluded.

The current study is the first to clearly define and focus on the measurement characteristics required for population surveillance (accuracy and sensitivity to change). There is only one other systematic review reporting on the measurement characteristics of self-report tools to measure SB [13], which concentrated on validity (assessed through rank correlation) and reliability, which are the measurement characteristics relevant to establishing association between SB and health. In agreement with the previous review, we found that the major flaw of most validation studies was the use of an inadequate criterion measure. The choice of criterion measure depends on the purpose of the tool. While direct observation should be considered the gold standard, if the purpose is to assess total sedentary time, then accurate postural sensors should be adequate (e.g. activPAL). In this review, only five out of 22 studies used an adequate criterion measure. Instead, many studies used an accelerometer which measures low levels of movement at the hip (e.g. ActiGraph) as a criterion measure, but such tools do not measure SB directly and can misclassify standing as sitting

For peer review only - http://bmjopen.bmj.com/site/about/guidelines.xhtml

[12]. Although it is possible that criterion measure may have provided a confounding effect on agreement (e.g. tools assessing previous day recall period (taxon 2.1, PAST, PAST-U) were only assessed against the activPAL, no clear trend towards better or worse agreement with a particular type of criterion measure or ActiGraph cut-off was apparent.

Despite the incomplete nature of the evidence, TASST enables the identification of desirable characteristics of self-report tools to measure SB when used for population surveillance. Firstly, tools assessing total SB time as a sum of behaviours (taxon 1.2.2.1; CHAMPS, LASA, MOST, PAST, PAST-U, SIT-Q-7d, STAQ) provided better accuracy than single item direct measurement (taxon 1.1.1; IPAQ-L-l7d, IPAQ-S-l7d and GPAQ) tools, especially when comparing tools with equivalent recall periods. However, this will be dependent on the behaviours or domains included within the sum, and whether they are exhaustive, consistent and mutually exclusive. Tools with a non-exhaustive sum will underestimate total time, for example, the Longitudinal Aging Study Amsterdam (LASA), found that a six item sum provided a better correlation with SB across the sample, but that a ten item sum was more accurate [33]. Conversely, tools which contain behaviours which might occur concurrently (such as watching TV and using a tablet computer) may lead to an over-estimate in total SB time [63]. Secondly, tools using a previous day recall period (taxon 2.1, PAST, PAST-U) tended to provide better accuracy than those with longer recall periods (taxa 2.2, 2.3 and 2.4). This corroborates recent research on the validity of computerised survey systems which assess SB using a past-day recall period [17, 66]. However, although tools using previous day recall may more accurate, it is likely that their sensitivity to change will be less good due to the higher underlying variability in daily SB [67].

For peer review only

Most tools currently used for population surveillance of SB systematically underestimate the amount of SB by two to four hours per day. Yet, self-report tools are still the most practical and economical means of population surveillance. Therefore, policy makers and clinicians should be aware that reports of population SB time are likely to be grossly underestimated, and should be cognisant of this fact when making decisions on implementing, developing and evaluating policy and public health interventions. In addition, policy makers and clinicians should be cautious in interpreting any reported difference in population SB time as a real change. The dearth of information about sensitivity to change of these tools means that we do not know the magnitude of change required to be certain that a change is real and not background variation. Moving forward, development of national and international surveillance systems should not be undertaken assuming that a tool is adequate because it has been used previously. Instead, investment should be made in research to evaluate the sensitivity to change and accuracy of tools to measure SB, paying attention to the potential trade-off between these two measurement characteristics. Such research should be carefully planned, to ensure that meaningful comparisons are investigated. The TASST taxonomy should be used as a useful framework to facilitate such a systematic approach.

For peer review only

Acknowledgements

The named authors present the study on behalf of the Seniors USP Team, which comprises: Dawn A Skelton (PI), Sebastien Chastin, Simon Cox, Elaine Coulter, Iva Čukić, Philippa Dall, Ian Deary, Geoff Der, Manon Dontje, Claire Fitzsimons, Catherine Gale, Jason Gill, Malcolm Granat, Cindy Gray, Carolyn Greig, Elaine Hindle, Karen Laird, Gillian Mead, Nanette Mutrie, Victoria Palmer, Ratko Radakovic, Naveed Sattar, Richard Shaw, John Starr, Sally Stewart, Sally Wyke. The authors would like to thank Calum Leask (CL) for his help in updating both of the searches in November 2016.

Competing Interests:

All authors had financial support from the Medical Research Council for the submitted work; no financial relationships with any organisations that might have an interest in the submitted work in the previous three years; no other relationships or activities that could appear to have influenced the submitted work.

Funding:

The study was performed in preparation for the Seniors USP: Understanding Sedentary Patterns project, which was supported by the Medical Research Council [MR/K025023/1]

Authors Contributions:

SC and DS conceived and supervised the study. PD and SC designed the study. PD, EC, CF and SC collected the data, and performed the systematic review. PD, CF and SC analysed the data for the

For peer review only

taxonomy. PD and SC analysed the data from the systematic review. All authors interpreted the data and critically revised the manuscript for important intellectual content.

Data Sharing: Data can be obtained from the corresponding author on request.

Figure Legends

Figure 1: TAxonomy of Self-reported Sedentary behaviour Tools (TASST)

Figure 2: PRISMA diagram of the validation systematic review

For peer review only

References

1. Kohl HW 3rd, Craig CL, Lambert EV, et al. The pandemic of physical inactivity: global action for public health. *Lancet* 2012;380:294–305.

2. Chastin S, Scwartz U, Skelton D. Development of a consensus taxonomy of sedentary behaviors (SIT): report of Delphi round 1. *PLoS One* 2013;8:e82313.

3. Sedentary Behaviour Research Network. Standardized use of the terms “sedentary” and “sedentary behaviours”. *Appl Physiol Nutr Metabol* 2012;37:540-2.

4. Healy GN, Matthews CE, Dunstan DW, Winkler EAH, Owen N. Sedentary time and cardio-metabolic biomarkers in US adults: NHANES 2003-06. *Eur Heart J* 2011;32:590-7.

5. Harvey JA, Chastin SFM, Skelton DA. How sedentary are older people? A systematic review of the amount of sedentary behavior. *J Aging Phys Act* 2015;23:471-87.

6. Proper KI, Singh AS, van Mechelen W, Chinapaw MJM. Sedentary behaviors and health outcomes among adults: a systematic review of prospective studies. *Am J Prev Med* 2011,40:174-82.

7. Thorp AA, Owen N, Neuhaus M, Dunstan DW. Sedentary behaviors and subsequent health outcomes in adults a systematic review of longitudinal studies, 1996-2011. *Am J Prev Med* 2011;41:207-15.

8. de Rezende LFM, Rey-López JP, Matsudo VKR, do Carmo Luiz O. Sedentary behavior and health outcomes among older adults: a systematic review. *BMC Public Health* 2014;14:333.

9. Dogra S, Stathokostas L. Sedentary behavior and physical activity are independent predictors of successful aging in middle-aged and older adults. *J Aging Res* 2012;2012:190654.

For peer review only

For peer review only - http://bmjopen.bmj.com/site/about/guidelines.xhtml

10. Department of Health. Start Active, Stay Active: A report on physical activity from the four home countries' Chief Medical Officers. 2011.

11. Guyatt G, Walter S, Norman G. Measuring change over time. assessing the usefulness of evaluative instruments. *J Chron Dis* 1987;40:171-8.

12. Kozey-Keadle S, Libertine A, Lyden K, Staudenmayer J, Freedson P. Validation of wearable monitors for assessing sedentary behavior. *Med Sci Sports Exerc* 2011;43:1561-7.

13. Healy GN, Clark BK, Winkler EAH, Gardiner PA, Brown WJ, Matthews CE. Measurement of adults' sedentary time in population-based studies. *Am J Prev Med* 2011,;41:216-27.

14. Bauman A, Ainsworth BE, Sallis JF, et al. The descriptive epidemiology of sitting: a 20 country comparison using the International Physical Activity Questionnaire (IPAQ). *Am J Prev Med* 2011;41:228-35.

15. Hekler EB, Buman MP, Haskell WL, et al. Reliability and validity of CHAMPS self-reported sedentary-vigorous intensity physical activity in older adults. *J Phys Act Health* 2012;9:225-36.

16. Chastin SFM, Culhane B, Dall PM. Questionnaire sitting time (IPAQ) compared to objective measurement via inclinometry (activPAL). *Physiol Meas* 2014;35:2319-28.

17. Kozey-Keadle S, Lyden K, Hickey A, et al. Validation of a previous day recall for measuring the location and purpose of active and sedentary behaviours compared to direct observation. *Int J Behav Nutr Phys Act* 2014;11:12.

18. Kmet LM, Lee RC, Cook LS. Standard quality assessment criteria for evaluating primary research papers from a variety of fields. *Edmonton:* Alberta Heritage Foundation for Medical Research 2004;1-22.

19. Banks E, Jorm L, Rogers K, Clements M, Bauman A. Screen-time, obesity, ageing and disability: findings from 91 266 participants in the 45 and Up Study. *Public Health Nutr* 2011;14:34-43.

For peer review only

20 Patel AV, Bernstein L, Deka A, et al. Leisure time spent sitting in relation to total mortality in a prospective cohort of US adults. *Am J Epidemiol* 2010;172:419-429.

21. Salmon J, Owen N, Crawford D, Bauman A, Sallis JF. Physical activity and sedentary behavior: a population-based study of barriers, enjoyment, and preference. *Health Psychol* 2003;22:178-88.

22. Gardiner PA, Healy GN, Eakin EG, et al. Associations between television viewing time and overall sitting time with the metabolic syndrome in older men and women: The Australian Diabetes Obesity and Lifestyle study. *JAGS* 2011;59:788-96.

23. Shields M, Tremblay MS. Sedentary behaviour and obesity. *Health Rep.* 2008;19:19-30.

24. Katzmarzyk PT, Church TS, Craig Cl, Bouchard C. Sitting time and mortality from all causes, cardiovascular disease, and cancer. *Med Sci Sports Exerc* 2009;41:998-1005.

25. Hamer M, Stamatakis E. Screen-based sedentary behaviour, physical activity, and muscle strength in the English Longitudinal Study of Ageing. *PLoS One* 2013;8:e66222.

26. Wareham NJ, Jakes RW, Rennie KL, Mitchell J, Hennings S, Day NE. Validity and repeatability of the EPIC-Norfolk physical activity questionnaire. *Int J Epidemiol* 2002;31:186-74.

27. Parsons TJ, Thomas C, Power C. Estimated activity patterns in British 45 year olds: cross-sectional findings from the 1958 British birth cohort. *Eur J Clin Nutr* 2009;63:978-85.

28. Armstrong T, Bull F. Development of the World Health Organization Global Physical Activity Questionnaire (GPAQ). *J Public Health* 2006;14:66-70.

29. Stamatakis E, Davis M, Stathi A, Hamer M. Associations between multiple indicators of objectively-measured and self-reported sedentary behaviour and cardiometabolic risk in older adults. *Prev Med* 2012;54:82-7.

For peer review only

30. Chau JY, Grunseit A, Midthjell K, et al. Sedentary behaviour and risk of mortality from all-causes and cardiometabolic diseases in adults: evidence from the HUNT3 population cohort. *Br J Sports Med* 2015;49:737-42.

31. Craig CL, Marshall AL, Sjöström M, et al. International Physical Activity Questionnaire: 12-country reliability and validity. *Med Sci Sports Exerc* 2003;35:1381-95.

32. Proper KI, Cerin E, Brown WJ, Owen N. Sitting time and socio-economic differences in overweight and obesity. *Int J Obes* 2007;31:169-76.

33. Visser M, Koster A. Development of a questionnaire to assess sedentary time in older persons – a comparative study using accelerometry. *BMC Geriatr* 2013;13:80.

34. Burazeri G, Goda A, Kark JD. Television viewing, leisure-time exercise and acute coronary syndrome in transitional Albania. *Prev Med* 2008;47:112-5.

35. Gardiner PA, Clark BK, Healy GN, Eakin EG, Winkler EAH, Owen N. Measuring older adults' sedentary time: reliability, validity, and responsiveness. *Med Sci Sports Exerc* 2011;43: 2127-33.

36. Ford ES. Combined television viewing and computer use and mortality from all-causes and diseases of the circulatory system among adults in the United States. *BMC Public Health* 2012;12:70

37. Evenson KR, McGinn AP. Test-retest reliability of adult surveillance measures for physical activity and inactivity. *Am J Prev Med* 2005;28:470-8.

38. Wolf AM, Hunter DJ, Colditz GA, et al. Reproducibility and validity of a self-administered physical activity questionnaire. *Int J Epidemiol* 1994;23:991-99.

39. Matthews CE, George SM, Moore SC, et al. Amount of time spent in sedentary behaviors and cause-specific mortality in US adults. *Am J Clin Nutr* 2012;ajcn-019620.

For peer review only

584 40. Salmon J, Bauman A, Crawford D, Timperio A, Owen N. The association between television viewing and overweight among Australian adults participating in varying levels of leisure-time physical activity. *Int J Obes Relat Metab Disord* 2000;24:600-6.

587 41. Washburn RA, McAuley E, Katula J, Mihalko SL, Boileau RA. The Physical Activity Scale for the Elderly (PASE): evidence for validity. *J Clin Epidemiol* 1999;52:643-51.

589 42. Clark BK, Winkler E, Healy GN, et al. Adults' past-day recall of sedentary time: reliability, validity and responsiveness. *Med Sci Sports Exerc* 2013;45:1198-207.

591 43. Clark BK, Pavey TG, Lim RF, Gomersall SR, Brown WJ. Past-day recall of sedentary time: validity of a self-reported measureof sedentary time in a university population. *J Sci Med Sport* 2016;19:237-41.

594 44. Balboa-Castillo T, León-Muñoz LM, Graciani A, Rodríguez-Artalejo F, Guallar-Castillón P. Longitudinal association of physical activity and sedentary behavior during leisure time with health-related quality of life in community-dwelling older adults. *Health Qual Life Outcomes* 2011;9:41-47.

597 45. Rosenberg DE, Norman GK, Wagner N, Patrick K, Calfas KJ, Sallis JF. Reliability and validity of the Sedentary Behaviour Questionnaire (SBQ) for adults. *J Phys Act Health* 2010;7:697-705.

599 46. Stamatakis E, Hamer M, Dunstan DW. Screen-based entertainment time, all-cause mortality, and cardiovascular events. *J Am Coll Cardiol* 2011;57:292-9.

601 47. Lynch BM, Friedenreich CM, KHandwala F, Liu A, Nicholas J, Csizmadi I. Development and testing of a past year measure of sedentary behaviour: the SIT-Q. *BMC Public Health*; 2014;14:899.

603 48. Wijndaele K, de Bourdeaudhuij I, Godino JG, et al. Reliability and validity of a domain-specific last 7-d sedentary time questionnaire. *Med Sci Sports Exerc* 2014;46:1248-60.

For peer review only

49. Csizmadi I, Neilson HK, Kopciuk KA, et al. The Sedentary Time and Activity reporting Questionnaire (STAR-Q): reliability and validity against doubly labelled water and 7-day activity diaries. *Am J Epidemiol* 2014;80:424-35.

50. Mensah K, Maire A, Oppert J-M, et al. Assessment of sedentary behaviours and transport-related activities by questionnaire: a validation study. *BMC Public Heath* 2016;16:753.

51. Dahl-Pederson KI, Hansen AW, Bjerregaard P, Jørgensen ME, Brage S. Validity of the international physical activity questionnaire in the arctic. *Med Sci Sports Exerc* 2013;45:728-36.

52. van Dyck D, Cardon G, Deforche B, de Bourdeaudhuij I. IPAQ interview version: convergent validity with accelerometers and comparison of physical activity and sedentary time levels with the self-administered version. *J Sports Med Phys Fitness* 2014;55:776-86.

53. Hagstromer M, Ainsworth BE, Sjostrom M. Comparison of a subjective and an objective measure of physical activity in a population sample. *J Phys Act Health* 2010;7:541-50.

54. Celis-Morales CA, Perez-Bravo F, Ibanez L, Salas C, Bailey MES, Gill JMR. Objective vs. self-reported physical activity and sedentary time: effects of measurement method on relationships with risk biomarkers. *PLoS ONE* 2012;7:e36345.

55. Wanner M, Probst-Hensch N, Kriemler S, Meier F, Autenrieth C, Martin BW. Validation of the long international physical activity questionnaire: influence of age and language region. *Prev Med Rep* 2016;3:250-6.

56. Dyrstad SM, Hansen BH, Holme IM, Anderssen SA. Comparison of self-reported versus accelerometer-measured physical activity. *Med Sci Sports Exerc* 2014;46:99-106.

57. Oyeyemi AL, Umar M, Oguche F, Aliyu SU, Oyeyemi AY. Accelerometer-determined physical activity and its comparison with the International Physical Activity Questionnaire in a sample of Nigerian adults. *PLoS ONE* 2014;9:e87233.

For peer review only

58. Hurtig-Wennlöf A, Hagströmer M, Olsson L. The International Physical Activity Questionnaire modified for the elderly: aspects of validity and feasibility. *Public Health Nutr* 2010;13:1847-54.

59. Grimm EK, Swartz AM, Hart T, Miller NE, Strath SJ. Comparison of the IPAQ-short form and accelerometry predictions of physical activity in older adults. *J Aging Phys Act* 2012;20:64-79.

60. Curry WB, Thompson JL. Comparability of Accelerometer- and IPAQ-derived physical activity and sedentary time in south Asian women: a cross-sectional study. *Eur J Sport Sci* 2015;15:655-62.

61. Alkahtani SA. Convergent validity: agreement between accelerometry and the Global Physical Activty Questionnaire in college-age Saudi men. *BMC Res Notes* 2016;9:436.

62. Gennuso KP, Matthews CE, Colbert LH. Reliability and validity of two self-report measures to assess sedentary behavior in older adults. *J Phys Act Health* 2015;12:727-32.

63. Busschaert C, de Bourdeaudhuij I, van Holle V, Chastin SFM, Cardon G, de Cocker K. Reliability and validity of three questionnaires measuring context-specific sedentary behaviour and associated correlates in adolescents, adults, and older adults. *Int J Behav Nutr Phys Act* 2015;12:117.

64. van Cauwenberg J, van Holle V, de Bourdeaudhuij I, Owen N, Deforche B. Older adults' reporting of specific sedentary behaviours: validity and reliability. *BMC Public Health* 2014;14:734.

65. Beaton DE, Bombardier C, Katz JN, Wright JG. A taxonomy for responsiveness. *J Clin Epidemiol* 2001;54:1204-71.

66. Gomersall SR, Pavey T, Clark BK, Jasman A, Brown WJ. Validity of a self-report recall tool for estimating sedentary behaviour in adults. *J Phys Act Health* 2015;12:1485-91.

67. Hart TL, Swartz AM, Cashin SE, Strath SJ. How many days of monitoring predict physical activity and sedentary behaviour in older adults? *Int J Behav Nutr Phys Act* 2011;8:62.

For peer review only

For peer review only - http://bmjopen.bmj.com/site/about/guidelines.xhtml

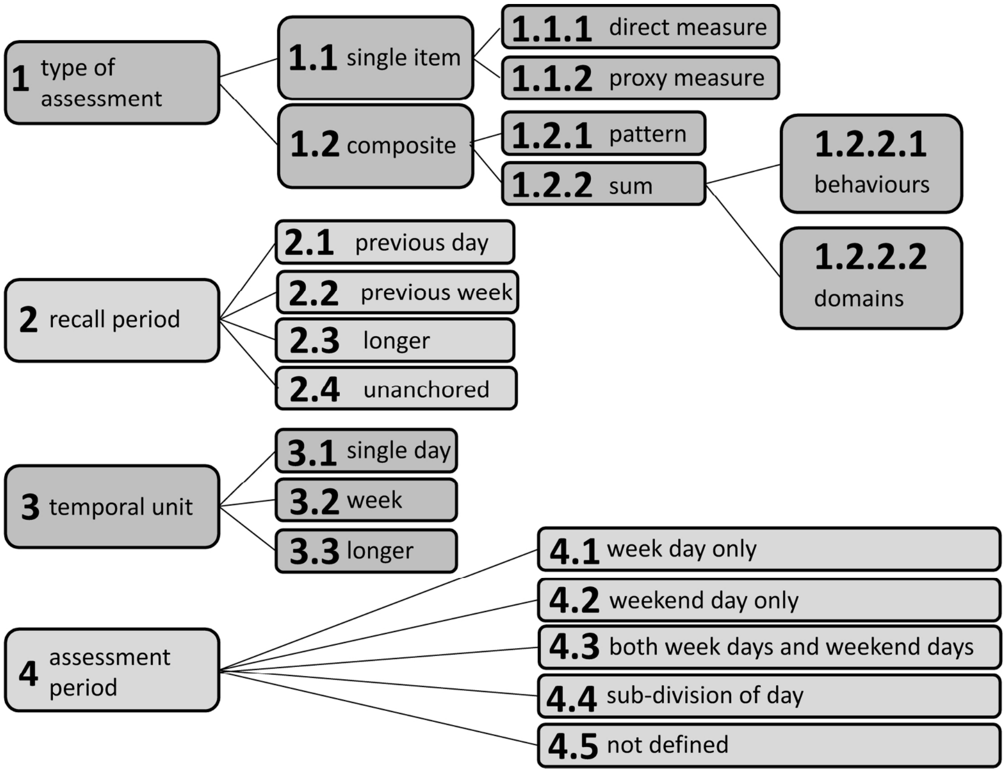


TAxonomy of Self-reported Sedentary behaviour Tools (TASST)
Figure 1
186x142mm (300 x 300 DPI)

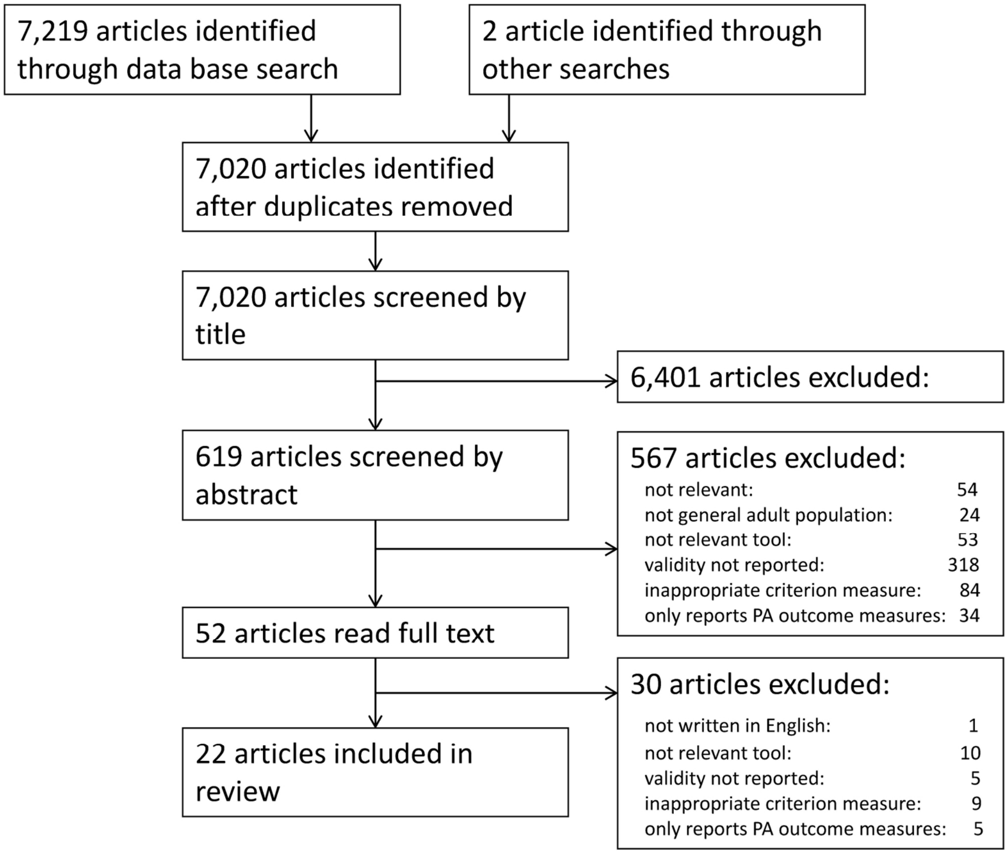


PRISMA diagram of the validation systematic review
Figure 2
171x144mm (300 x 300 DPI)

For peer review only - http://bmjopen.bmj.com/site/about/guidelines.xhtml

# PRISMA 2009 Checklist

| Section/topic | # | Checklist item | Reported on page # |
|---|---|---|---|
| **TITLE** | | | |
| Title | 1 | Identify the report as a systematic review, meta-analysis, or both. | 1 |
| **ABSTRACT** | | | |
| Structured summary | 2 | Provide a structured summary including, as applicable: background; objectives; data sources; study eligibility criteria, participants, and interventions; study appraisal and synthesis methods; results; limitations; conclusions and implications of key findings; systematic review registration number. | 2 |
| **INTRODUCTION** | | | |
| Rationale | 3 | Describe the rationale for the review in the context of what is already known. | 3 |
| Objectives | 4 | Provide an explicit statement of questions being addressed with reference to participants, interventions, comparisons, outcomes, and study design (PICOS). | 3 |
| **METHODS** | | | |
| Protocol and registration | 5 | Indicate if a review protocol exists, if and where it can be accessed (e.g., Web address), and, if available, provide registration information including registration number. | 4 |
| Eligibility criteria | 6 | Specify study characteristics (e.g., PICOS, length of follow-up) and report characteristics (e.g., years considered, language, publication status) used as criteria for eligibility, giving rationale. | 4 & 5 |
| Information sources | 7 | Describe all information sources (e.g., databases with dates of coverage, contact with study authors to identify additional studies) in the search and date last searched. | 4 & 5 |
| Search | 8 | Present full electronic search strategy for at least one database, including any limits used, such that it could be repeated. | 5 |
| Study selection | 9 | State the process for selecting studies (i.e., screening, eligibility, included in systematic review, and, if applicable, included in the meta-analysis). | 5 |
| Data collection process | 10 | Describe method of data extraction from reports (e.g., piloted forms, independently, in duplicate) and any processes for obtaining and confirming data from investigators. | 5 |
| Data items | 11 | List and define all variables for which data were sought (e.g., PICOS, funding sources) and any assumptions and simplifications made. | 5 |
| Risk of bias in individual studies | 12 | Describe methods used for assessing risk of bias of individual studies (including specification of whether this was done at the study or outcome level), and how this information is to be used in any data synthesis. | Not reporting on trials, so not done |

For peer review only

# PRISMA 2009 Checklist

| | | | |
|---|---|---|---|
| Summary measures | 13 | State the principal summary measures (e.g., risk ratio, difference in means). | 5 |
| Synthesis of results | 14 | Describe the methods of handling data and combining results of studies, if done, including measures of consistency (e.g., $I^2$) for each meta-analysis. | Not done |



| Section/topic | # | Checklist item | Reported on page # |
|---|---|---|---|
| Risk of bias across studies | 15 | Specify any assessment of risk of bias that may affect the cumulative evidence (e.g., publication bias, selective reporting within studies). | 9 |
| Additional analyses | 16 | Describe methods of additional analyses (e.g., sensitivity or subgroup analyses, meta-regression), if done, indicating which were pre-specified. | Not done |
| **RESULTS** | | | |
| Study selection | 17 | Give numbers of studies screened, assessed for eligibility, and included in the review, with reasons for exclusions at each stage, ideally with a flow diagram. | 8, figure 2 |
| Study characteristics | 18 | For each study, present characteristics for which data were extracted (e.g., study size, PICOS, follow-up period) and provide the citations. | Table 3 |
| Risk of bias within studies | 19 | Present data on risk of bias of each study and, if available, any outcome level assessment (see item 12). | 8 |
| Results of individual studies | 20 | For all outcomes considered (benefits or harms), present, for each study: (a) simple summary data for each intervention group (b) effect estimates and confidence intervals, ideally with a forest plot. | Table 3, pages 9 & 10 (not a review of interventions) |
| Synthesis of results | 21 | Present results of each meta-analysis done, including confidence intervals and measures of consistency. | Not enough data for meta-analysis |
| Risk of bias across studies | 22 | Present results of any assessment of risk of bias across studies (see Item 15). | 9 |
| Additional analysis | 23 | Give results of additional analyses, if done (e.g., sensitivity or subgroup analyses, meta-regression [see Item 16]). | Not done |
| **DISCUSSION** | | | |
| Summary of evidence | 24 | Summarize the main findings including the strength of evidence for each main outcome; consider their relevance to key groups (e.g., healthcare providers, users, and policy makers). | 12 |
| Limitations | 25 | Discuss limitations at study and outcome level (e.g., risk of bias), and at review-level (e.g., incomplete retrieval of identified research, reporting bias). | 11 & 12 |

For peer review only - http://bmjopen.bmj.com/site/about/guidelines.xhtml

# PRISMA 2009 Checklist

| | | | |
|---|---|---|---|
| Conclusions | 26 | Provide a general interpretation of the results in the context of other evidence, and implications for future research. | 12 |
| **FUNDING** | | | |
| Funding | 27 | Describe sources of funding for the systematic review and other support (e.g., supply of data); role of funders for the systematic review. | 14 |

*From:* Moher D, Liberati A, Tetzlaff J, Altman DG, The PRISMA Group (2009). Preferred Reporting Items for Systematic Reviews and Meta-Analyses: The PRISMA Statement. PLoS Med 6(6): e1000097. doi:10.1371/journal.pmed1000097

For more information, visit: www.prisma-statement.org.

Page 2 of 2

For peer review only

BMJ Open

# The TAxonomy of Self-reported Sedentary behaviour Tools (TASST) framework for development, comparison and evaluation of self-report tools: content analysis and systematic review.

For peer review only

| | |
|---|---|
| Journal: | *BMJ Open* |
| Manuscript ID | bmjopen-2016-013844.R2 |
| Article Type: | Research |
| Date Submitted by the Author: | 30-Jan-2017 |
| Complete List of Authors: | Dall, Philippa; Glasgow Caledonian University School of Health and Life Sciences, Department of Psychology, Social Work & Allied Health Sciences<br>Coulter, Elaine; University of Glasgow<br>Fitzsimons, Claire F.; Univ Edinburgh, ISPEHS<br>Skelton, Dawn; Glasgow Caledonian University, School of Health & Life Sciences<br>Chastin, Sebastian; Glasgow Caledonian University |
| <b>Primary Subject Heading</b>: | Public health |
| Secondary Subject Heading: | Epidemiology, Research methods, Sports and exercise medicine |
| Keywords: | sedentary behaviour, sitting, population surveillance, measurement, validation |
| | |

SCHOLARONE™ Manuscripts

**Title:** The TAxonomy of Self-reported Sedentary behaviour Tools (TASST) framework for development, comparison and evaluation of self-report tools: content analysis and systematic review.

**Authors:**

Dall PM[1], Coulter EH[2], Fitzsimons CF[3], Skelton DA[1], Chastin SFM[1], on behalf of the Seniors USP Team.

[1] Institute for Applied Health Research, Glasgow Caledonian University, Cowcaddens Road, Glasgow, G4 0BA

[2] Department of Nursing and Healthcare, Glasgow University, 59 Oakfield Avenue, Glasgow, G12 8LL

[3] Sport, Physical Education and Health Sciences Institute, Edinburgh University, St Leonard's Land, Holyrood Road, Edinburgh, EH8 8AQ

corresponding author:

Dr Philippa Dall

School of Health & Life Sciences

Glasgow Caledonian University

Cowcaddens Road

Glasgow

G4 0BA

philippa.dall@gcu.ac.uk

Word count: 5,107

For peer review only

**ABSTRACT**

**Objective:** Sedentary behaviour (SB) has distinct deleterious health outcomes, yet there is no consensus on best practice for measurement. This study aimed to identify the optimal self-report tool for population surveillance of SB, using a systematic framework.

**Design:** A framework, TAxonomy of Self-report SB Tools (TASST), consisting of four domains (type of assessment, recall period, temporal unit, and assessment period), was developed based on a systematic inventory of existing tools. The inventory was achieved through a systematic review of studies reporting SB and tracing back to the original description. A systematic review of the accuracy and sensitivity to change of these tools was then mapped against TASST domains.

**Data Sources:** Systematic searches were conducted via EBSCO, reference lists and expert opinion.

**Eligibility Criteria for selecting studies:** The inventory included tools measuring SB in adults that could be self-completed at one sitting, and excluded tools measuring SB in specific populations or contexts. The systematic review included studies reporting on the accuracy against an objective measure of SB and/or sensitivity to change of a tool in the inventory.

**Results:** The systematic review initially identified 32 distinct tools (141 questions), which were used to develop the TASST framework. Twenty-two studies evaluated accuracy and/or sensitivity to change representing only 8 taxa. Assessing SB as a sum of behaviours and using a previous day recall were the most promising features of existing tools. Accuracy was poor for all existing tools, with both under and over estimation of SB. There was a lack of evidence about sensitivity to change.

**Conclusions:** Despite the limited evidence, mapping existing SB tools onto the TASST framework has enabled informed recommendations to be made about the most promising features for a surveillance tool, identified aspects on which future research and development of SB surveillance tools should focus.

For peer review only - http://bmjopen.bmj.com/site/about/guidelines.xhtml

**Systematic Review Registration**

PROSPERO/CRD42014009851

**KEY WORDS:**

sedentary behaviour;  sitting; population surveillance; measurement; validation

**STRENGTHS AND LIMITATIONS OF THIS STUDY:**

- A systematic approach was taken towards classifying self-reported measures of sedentary behaviour, allowing a structured approach to measurement in the future
- An example of use of the framework is presented, mapping accuracy and sensitivity to change of self-report sedentary behaviour measures on to the framework
- Although designed to be generic, the TASST framework was developed excluding tools measuring sedentary behaviour in specialised populations and contexts, e.g. children or the workplace, and the framework may therefore not cover some aspects of these tools
- There is the potential for a language bias, as full-text articles not in English were not included in the systematic reviews.

For peer review only

## BACKGROUND

Physical inactivity is currently at pandemic levels [1] and is a global public health concern. Sedentary behaviour (SB), an umbrella term for all waking time spent in non-exercising sitting or reclining postures [2, 3] such as sitting during work, motorised transport or watching TV, is the largest contributor to inactivity [4,5]. Higher levels of SB have been associated with poor physical and mental health, increased risk of chronic disease and less successful ageing [6-9]. Consequently, several countries, including the UK, have issued recommendations to reduce SB at all ages as part of their national physical activity guidelines [10]. Population surveillance is urgently needed to monitor the impact of such policy, track changes in SB over time, and to evaluate public health interventions targeting SB. In order to provide effective surveillance upon which to base future policy decisions, such surveillance tools should be accurate (provide a true measure of the actual amount of SB in a population) and sensitive to change (provide the true difference in SB between two measurement time points) [11].

Objective body worn sensors, that measure posture, demonstrate good accuracy for measuring total duration of SB against the gold standard of direct observation [12], but they are expensive and challenging to use for population surveillance. Self-report tools provide a pragmatic choice for population surveillance and have the potential to provide context rich information, useful for intervention development [13]. To date, surveys assessing SB have predominantly used self-report tools [14], which are generally adapted from tools not specifically designed to measure that behaviour (e.g. tools designed to measure physical activity) [15], and which have not been evaluated for population surveillance purposes [14]. No framework currently exists with which to describe and compare SB self-report tools, meaning there is currently no way of systematically selecting an appropriate tool. A previous systematic review of the measurement characteristics of self-report tools measuring SB, reported acceptable to good reliability but low to moderate correlation with a

For peer review only

(non-gold standard) criterion measure [13]. This suggests that self-report measures of SB are acceptable tools to establish epidemiological evidence of an association between SB and health [13]. However, it is possible that the scale of the problem may be vastly underestimated, as differences of 2-4 hours per day (approximately 20% of SB) have been reported between self-report and objective tools [16].

The primary aim of this study was to identify, in a systematic manner, the optimal self-report tool to measure SB for use in population surveillance. Although self-report SB tools can and will be used in other areas of research, this study focussed on population surveillance as an area that is crucial to the development of public health policy. To fulfil the primary aim, a framework was created to describe the features of self-report tools measuring SB, the TAxonomy of Self-report Sedentary behaviour Tools (TASST). A systematic inventory of existing self-report tools to measure SB was mapped onto TASST, and the measurement characteristics of these tools, focussing on accuracy and sensitivity to change, were evaluated, with explicit reference to the domains of the taxonomy framework.

## METHODS

The study protocol (PROSPERO CRD42014009851), was conducted in three phases. In phase 1 an exhaustive inventory of self-reported tools to measure SB in adults and older adults was established using a structured search protocol. Phase 2 was the development of a taxonomy based on content analysis of the items and questions in the tools. In phase 3, a systematic literature review of the measurement characteristics of the tools in the inventory was conducted and mapped onto the taxonomy.

For peer review only

**Phase 1: Systematic inventory of self-report tools**

The aim of the systematic inventory was to compile an exhaustive list of self-report tools which could be used to measure SB in adults (≥18 years) and older adults (≥60 years). Since the aim was to identify tools and not to identify articles, this stage does not have the same methodology as a systematic literature review. A literature search was conducted in October 2013 (updated November 2016), for articles reporting SB as an outcome measure. From this review, a list of self-report tools which measured SB was compiled. References lists were reviewed and experts consulted to identify any additional tools to include in the inventory. The inventory then was consolidated to amalgamate tools referred to by different names, and to trace back to the original version. Articles which added significant new questions to tools were included as a separate tool. We defined significant new questions to be at least one question which added or changed the type of sedentary behaviour or the time period considered by the tool. Changes in phrasing of the question were not considered sufficient to be considered as a separate tool. Tools used in a single study and those without names/acronyms were included as separate tools.

To be included in the inventory, tools had to: be suitable for use for large scale population studies of adults or older adults, including being suitable for self-completion by the respondent at a single point in time (a pragmatic requirement to minimise participant burden); and measure SB or a proxy measure of SB (e.g. TV viewing). Although there is great interest in the sedentary behaviour across many populations and contexts, for pragmatic purposes initial taxonomy development was limited to a core of self-report tools widely applicable to the general adult population. Therefore, tools were excluded from the inventory: if they were designed specifically to assess SB in children or other specialised populations (e.g. medical conditions); if they were designed specifically to assess SB in a specialised context, (e.g. workplace or care settings); if continuous reporting over extended periods of time was required (e.g. diaries or time-use surveys); or if significant interviewer interactions were

For peer review only

required. Self-report tools that could be administered by telephone or interview were not automatically excluded, however tools such as the PDR (Previous Day Recall) [17], in which the interviewer works through lists of several hundred items, were excluded.

**Phase 2: Development of a taxonomy**

Only tools identified in the initial search were used to develop the taxonomy. The original text was extracted for each question relating to SB in each of the self-report tools identified in the inventory. Content analysis was conducted on the text to extract all of the attributes in the questions that were used to describe and constrain what aspect of SB was measured by that question. For example, in the question "During the last 7 days, how much time did you usually spend sitting on a week day?", attributes extracted relating to the measurement of SB would be "during the last 7 days", "time spent sitting" and "on a week day". Attributes were then grouped into mutually exclusive domains covering similar aspects of measurement, and categories within those domains were defined iteratively. A new category was created each time a tool did not fit within an existing category. The full taxonomy was then assembled and streamlined by merging categories with overlapping meaning. Finally, consideration was given to potential future developments of self-report tools to measure SB, such as the growing interest in the pattern of accumulation of sedentary behaviour, by adding any categories to the taxonomy considered useful in the future. The resulting taxonomy was then tested by ensuring that all tools could be classified similarly by two independent researchers and that the taxonomy fully defined the tool.

**Phase 3: Systematic review of measurement characteristics**

Finally, a systematic literature search in relevant health databases was conducted in December 2014 (updated November 2016) via EBSCO host. The search combined the name of the tool including

For peer review only

variants and acronyms (except where the acronym was also a common word, e.g. PAST, MOST), with search terms relating to measurement characteristics (valid* /reliab* /repons* /sensitiv* /calibrat* /accura* /agreement /psychometric* /clinimetric* /"measurement characteristics" /Reliability and Validity (MeSH)). Articles were included only if they reported in English on the accuracy of a tools in the inventory against an objective criterion measure of SB, and/or sensitivity to change. Although articles were only included in the review if they assessed accuracy or sensitivity to change, the search terms included a wide range of psychometric properties in order to maximise the chances of finding eligible articles.

Exclusion by title, then abstract, then full-text was conducted by two researchers from a pool of five [PD, EC, CF, SC, CL]. In the case of disagreement, the article was carried forward in to the next round, or at full-text stage a third researcher was consulted to ensure consensus. Data (tool, criterion, population, statistical analysis, accuracy of sedentary behaviour, sensitivity to change of sedentary behaviour) was extracted and quality was assessed independently by two researchers from a pool of three [PD, CF, SC]. Disagreements were resolved by discussion. Quality was assessed using QUALSYST [18], modified to include an additional item for the criterion measure. As per the QUALSYST guidelines, the quality score for the article (range 0-1) was used to identify common methodological strengths and flaws, rather than as an objective representation of high/low quality. Accuracy and sensitivity to change extracted from included articles were reported for tools in relation to the TASST taxonomy.

## RESULTS

### Inventory

For peer review only

The systematic inventory identified 37 distinct self-report tools used to measure SB in adults and older adults, 32 of which were identified in the initial search and used to form the taxonomy (Table 1). The International Physical Activity Questionnaire (IPAQ) was originally developed with four different versions, which were included separately in the inventory (combinations of the long and short versions, and last seven days and usual week recall). The 45 and Up study asked different questions in its baseline and follow-up questionnaires, which have been included as separate tools. Three tools, termed "modified" versions, were included where questions had been added or modified to the original tool (EPAQ2, NHANES, and IPAQ-L, representing a 5$^{th}$ version of the IPAQ in the inventory), and were considered to form a substantially different version. Some tools identified were used in only a single study, and these were included in the inventory, referred to by the study name. The 32 tools in the original inventory comprised of 141 individual questions, consisting of between 1 and 20 questions per tool. An evaluation of the content of these individual items formed the basis of the TASST taxonomy.

For peer review only

199 Table 1 Tools measuring SB for population surveillance identified in the inventory

| Acronym | Name of Tool/Study | Key reference |
|---|---|---|
| 45Up-B | 45 and Up study, baseline questionnaire | [19] |
| 45Up-F | 45 and Up study, follow up questionnaire | [19] |
| ACS2 | American Cancer Society, Cancer Prevention Study cohort II | [20] |
| ALTS | Australian Leisure Time Sitting questionnaire | [21] |
| AusDiab | The Australian Diabetes Obesity and Lifestyle study | [22] |
| CCHS | Canadian Community Health Survey | [23] |
| CFS | Canadian Fitness Survey | [24] |
| CHAMPS | Community Health Activities Model Program for Seniors physical activity questionnaire | [15] |
| ELSA | English Longitudinal Study of Ageing | [25] |
| EPAQ2 | European Prospective Investigation of Cancer (EPIC)-Norfolk Physical Activity Questionnaire | [26] |
| mod EQPAQ2 | modified version of the EPIC-Norfolk Physical Activity Questionnaire | [27] |
| GPAQ | Global Physical Activity Questionnaire | [28] |
| HSE | Health Survey for England | [29] |
| HUNT3 | Nord-Trøndelag Health Study 3 | [30] |
| IPAQ-L l7d | International Physical Activity Questionnaire, Long version, last 7 days | [31] |
| IPAQ-L uw | International Physical Activity Questionnaire, Long version, usual week | [31] |
| mod IPAQ-L | modified version of the International Physical Activity Questionnaire, Long version | [32] |
| IPAQ-S l7d | International Physical Activity Questionnaire, Short version, last 7 days | [31] |
| IPAQ-S uw | International Physical Activity Questionnaire, Short version, usual week | [31] |
| LASA | Longitudinal Aging Study Amsterdam | [33] |
| MLTPAQ | Minnesota Leisure Time Physical Activity Questionnaire | [34] |
| MOST | Measuring Older adults' Sedentary Time questionnaire | [35] |
| NHANES | National Health and Nutrition Examination Survey | [36] |
| mod NHANES | modified version of the National Health and Nutrition Examination Survey | [37] |
| NHS2 | Nurses Health Survey II | [38] |
| NIH-AARP DHS | National Institutes of Health – American Association of Retired Persons (NIH-AARP) Diet and Health Survey | [39] |
| NSWPAS | New South Wales Physical Activity Survey | [40] |
| PASE | Physical Activity Scale for the Elderly | [41] |
| PAST | Past-day Adults Sedentary Time questionnaire | [42] |
| PAST-U* | Past-day Adults Sedentary Time questionnaire – University version | [43] |
| PCSpa | prospective cohort study (Spain) | [44] |
| SBQ | Sedentary Behaviour Questionnaire | [45] |
| SHS | Scottish Health Survey | [46] |
| SIT-Q* | SIT-Q | [47] |
| SIT-Q-7d* | past seven day version of the SIT-Q | [48] |
| STAR-Q* | Sedentary Time and Reporting Questionnaire | [49] |
| STAQ* | Sedentary, Transportation and Activity Questionnaire | [50] |

For peer review only - http://bmjopen.bmj.com/site/about/guidelines.xhtml

Acronym: the commonly used acronym of the tool, or the short identifier adopted for this article. Name of Tool: either the name of the tool, or the name of the single study using these questions/tool. Key reference: references provided here are not exhaustive, but refer either to an early or well cited description of the tool, or the study in which the tool was used or adapted. Tools marked with an asterisk (*) were identified in the updated search, and were not used to create the taxonomy

For peer review only

**TAxonomy for Self-report Sedentary behaviour Tools (TASST)**

The taxonomy derived from the inventory of self-report tools to measure SB (Figure 1) comprises of four domains, which characterise different aspects of the tool: type of assessment, recall period, temporal unit, and assessment period. All four aspects are required to describe the tool. Within each aspect, the taxonomy functions as a tree, meaning you can identify a single end point (taxon) which fully describes each question in a tool.

The type of assessment domain of the taxonomy covers the way that the outcome of time spent in SB is derived from the tool. Tools can either ask about a single aspect of SB (1.1 single item), or a composite aspect (1.2 composite). Tools using a single item of assessment will generate all of their information about SB within the relevant period of assessment in a single question. That single item can either ask about sitting time directly (1.1.1 direct measure) or it can ask about a single behaviour related to SB which is then used as a proxy measure of SB duration (1.1.2 proxy measure). Composite items of assessment ask multiple questions about several aspects of SB for the same period of assessment. One form of composite item would be to ask about the pattern (i.e. frequency and timing) of SB accumulated throughout the recall period (1.2.1 pattern). However, the most common form of composite item is created as a sum (1.2.2 sum) of the time spent in SB in a range of different activities or situations. The sum can either be formed from questions asking about specific behaviours (1.2.2.1), activities such as TV viewing, hobbies, talking with friends, or they can be based on domains (1.2.2.2), locations or situations where you can sit, such as at home, for transport and at work.

The recall period is total time over which the respondent is asked to consider their SB when answering the questions. The recall period can be anchored to the present time in which case it

For peer review only - http://bmjopen.bmj.com/site/about/guidelines.xhtml

refers to a specific length of time prior to now, for example yesterday (2.1 previous day), last week (2.2 previous week), or a longer period such as the last month or year (2.3 longer). The recall period can also be unanchored (2.4), in which case the respondent is not asked about a specific period but is asked about a general period of time, for example asking about SB in a typical week.

The temporal unit is the duration within the recall period that a respondent is asked to report their SB for. For example, in the question “on a typical day last week, how long did you sit?” the recall period is the previous week, but the temporal unit is a day. Within the taxonomy, the temporal units may be a day (3.1), a week (3.2) or longer (3.3). Within a particular recall period, it is possible to have any temporal unit that is of identical or shorter duration than the recall period.

The period of assessment is completed by identifying any specific restrictions that are placed on the type of temporal unit recalled. The categories within the assessment period domain clarify whether a respondent is asked questions regarding a particular type of day, for example only about week days (4.1), only weekend days (4.2), or is asked about weekdays and weekend days in separate questions (4.3 both). Additionally, the assessment period domain can identify if a respondent is asked about particular sub divisions of the day (4.4) in separate questions, for example time spent sitting before 6pm. The final taxon in the assessment period is termed ‘not defined’ (4.5), this represents the situation where a respondent is asked about all temporal units (e.g. days) within the recall period (e.g. last week) without any specific distinction being made between them. It is a global category, which usually represents a decision not to separate out these categories, as opposed to a failure to define this domain.

**Mapping the Inventory on to the Taxonomy**

For peer review only - http://bmjopen.bmj.com/site/about/guidelines.xhtml

The 37 tools identified in the inventory were mapped against the TASST taxonomy (Table 2). Approximately half of the tools in the inventory (n=17) used a single item of assessment, thirteen used a direct measure and seven used a proxy measure. Three tools (45Up-B, AusDiab, NIH-AARP DHS) asked single item questions about both a direct measure and a proxy measure, but not in a manner in which they could be used as a sum, and have therefore been included in the count for both taxa. Proxy measures were predominantly based on TV viewing (n=5). Twenty tools used composite assessment, all of which used a sum as that composite item. The vast majority of sums were formed from questions asking about different behaviours (n=19), with only one sum formed from questions asking about different domains. The tools using a sum of behaviours generally included the common proxy measures of TV viewing (n=19) and computer use (n=17) within the sum. Many tools included questions for behaviours based on leisure pursuits (n=14), in social contexts (n=9), and during transportation (n=13). Often several behaviours of each type were considered in separate questions (e.g. asking about time sitting while reading separately from time spent sitting listening to music). Questions based on time working were included in ten tools, but were explicitly excluded in four tools. Less frequently, tools included questions based on rest (n=5), or used an "other" category to cover circumstances not explicit within the questions (n=7).

For peer review only

For peer review only - http://bmjopen.bmj.com/site/about/guidelines.xhtml

Table 2: Mapping of the tools measuring SB identified in the inventory onto the TASST taxonomy.

| Taxonomy Item | | N | Tools | Accuracy | Sensitivity to change |
|---|---|---|---|---|---|
| **1** | **Type of Assessment** | | | | |
| 1.1 | Single item | 17 | | **Underestimate with large systematic and a random error** | + |
| 1.1.1 | Direct measure | 13 | 45Up-B; ACS2; AusDiab; CFS; GPAQ; HUNT3; IPAQ-L l7d; IPAQ-L uw; IPAQ-S l7d; IPAQ-S uw; NIH-AARP DHS; PASE; PCSPa | | |
| 1.1.2 | Proxy measure | 7 | 45Up-B; AusDiab; ELSA; MLTPAQ; NIH-AARP DHS; NSWPAS; SHS | | |
| 1.2 | Composite item | 20 | | **Smaller systematic error but there is a potential to overestimate** | + |
| 1.2.1 | Pattern | 0 | | | |
| 1.2.2 | Sum | | | | |
| 1.2.2.1 | Behaviours | 19 | 45Up-F; ALTS; CCHS; CHAMPS; EPAQ2; mod EPAQ2; HSE; mod IPAQ-L; LASA; MOST; NHANES; mod NHANES; PAST; PAST-U; SBQ; SIT-Q; SIT-Q-7d; STAR-Q; STAQ | | |
| 1.2.2.2 | Domains | 1 | NHS2 | | |
| **2** | **Recall period** | | | | |
| 2.1 | Previous day | 2 | PAST; PAST-U | **+** | - |
| 2.2 | Previous week | 9 | 45Up-F; ALTS; AusDiab; IPAQ-L l7d; IPAQ-S l7d; mod IPAQ-L; MOST; PASE; SIT-Q-7d | **-** | + |
| 2.3 | Longer | 11 | ACS2; CCHS; CHAMPS; EPAQ2; mod EPAQ2; HSE; NHANES; NIH-AARP DHS; SIT-Q; STAR-Q; STAQ | **-** | - |
| 2.4 | Unanchored | 15 | 45Up-B; CFS; ELSA; GPAQ; HUNT3; IPAQ-L uw; IPAQ-S uw; LASA; MLTPAQ; mod NHANES; NHS2; NSWPAS; PCSpa; SBQ; SHS | | |
| **3** | **Temporal Unit** | | | | |
| 3.1 | Day | 32 | 45Up-B; 45Up-F; ACS2; AusDiab; CFS; ELSA; EPAQ2; mod EPAQ2; GPAQ; HSE; HUNT3; IPAQ-L l7d; IPAQ-L uw; IPAQ-S l7d; IPAQ-S uw; mod IPAQ-L; LASA; MLTPAQ; NHANES; mod NHANES; NIH-AARP DHS; NSWPAS; PASE; PAST; PAST-U; PCSpa; SBQ; SHS; SIT-Q; SIT-Q-7d; STAR-Q; STAQ | **+** | + |
| 3.2 | Week | 5 | ALTS; CCHS; CHAMPS; MOST; NHS2 | - | - |
| 3.3 | Longer | 0 | | - | - |
| **4** | **Assessment Period** | | | | |
| 4.1 | Weekdays only | 2 | IPAQ-S l7d; IPAQ-S uw | - | + |
| 4.2 | Weekend days only | 0 | | - | - |
| 4.3 | Both weekdays and weekend days | 14 | 45Up-F; AusDiab; ELSA; HSE; IPAQ-L l7d; IPAQ-L uw; mod IPAQ-L; LASA; NSWPAS; PCSpa; SBQ; SHS; SIT-Q-7d; STAQ | + | - |
| 4.4 | Subdivision of the day | 1 | EPAQ2 | + | - |
| 4.5 | Not defined | 21 | 45Up-B; ACS2; ALTS; CCHS; CFS; CHAMPS; EPAQ2; mod EPAQ2; GPAQ; HUNT3; MLTPAQ; MOST; NHS2; NHANES; mod NHANES; NIH-AARP DHS; PASE; PAST; PAST-U; SIT-Q; STAR-Q | **Better for older adults** | + |

Full names for the acronyms reported in the Tools column can be found in Table 1. Recommendations in bold are backed by evidence from the systematic review. Recommendations which are not bold are theoretical but no evidence could be found in the literature. + represents a positive attribute; – a negative attribute.



For peer review only - http://bmjopen.bmj.com/site/about/guidelines.xhtml

A little under half of the tools in the inventory used an unanchored recall period (n=15), nine used a previous week recall period, and eleven used a longer recall period. Only two tools (PAST, PAST-U) in the inventory used a previous day recall period. The majority of tools used a temporal unit of a day (n=32), with five (ALTS, CCHS, CHAMPS, MOST, NHS2) using a temporal unit of a week. A single question within the EPAQ2 questionnaire was based on a temporal unit longer than a week, but the other three questions in that tool were based on a temporal unit of a day. Just over half the tools (n=21) did not define specific days or time periods in their questions, but asked about the temporal unit within the recall period as a single entity. Sixteen tools used questions specifically referring to week or weekend days, fourteen asking about both week and weekend days, while two asked only about week days. Only one tool (EPAQ2) referred to specific sub-divisions of the day in their questions.

**Systematic search for measurement characteristics**

The systematic search returned 7,221 references, and after removal of duplicate and assessment against exclusion criteria (>99% agreement between reviewers), a total of 22 studies were included in the review (figure 2, table 3).

For peer review only

Table 3: Measurement characteristics of tools measuring SB, presented by tool and taxon

| Tool | Taxon (refer to figure 1) | N | Population *(Country)* | Criterion measure (definition of SB) | QUALSYST Score | Agreement (*hours/day*) tool - criterion [limit of agreement] | Sensitivity to change | Ref |
|---|---|---|---|---|---|---|---|---|
| IPAQ-Long l7d | 1.1.1/2.2/3.1/4.3 | 1508 | A & OA (Greenland) | actiHeart (<1.5MET) | 0.67 | -3.0 [not reported] for adults<br>-6.0 [not reported] for older adults | -- | [51] |
| | | 542 | A (Netherlands) | Actigraph (<100 count/min) | 0.78 | -1.6 [-6.4 3.2] | -- | [52] |
| | | 980 | A (Sweden) | Actigraph (<100 count/min) | 0.67 | +2.2 [-4.5 9.5] | -- | [53] |
| | | 69 | A (UK) | activPAL (sitting/lying postures) | 0.78 | -2.2 [-7.2 3.7] | -- | [16] |
| | | 317 | A (Chile) | Actigraph (<100 count/min) | 0.78 | -1.1 [-3.8 1.5] | -- | [54] |
| | | 346 | A & OA (Switzerland) | ActiGraph (<150 count/min) | 0.78 | -3.8 [-9.3 1.7] | -- | [55] |
| IPAQ-Short l7d | 1.1.1/2.2/3.1/4.1 | 1751 | A & OA (Norway) | Actigraph (<100 count/min) | 0.67 | -1.8 [not reported] for adults<br>+3.5 [not reported] for older adults | -- | [56] |
| | | 144 | A (Nigeria) | Actigraph (<100 count/min) | 0.78 | -3.0 [-8.5 2.5] | -- | [57] |
| | | 54 | OA (Sweden) | Actigraph (<100 count/min) | 0.56 | -1.5 [not reported] | -- | [58] |
| | | 127 | OA (USA) | Actigraph (<50 count/min) | 0.72 | -4.4 [-10.0 -1.4] | -- | [59] |
| | | 50 | A & OA (UK) | Actigraph (<50 count/min) | 0.72 | -0.5 [-1.9 0.8] | -- | [60] |
| GPAQ | 1.1.1/2.4/3.1/4.5 | 62 | A (Saudi Arabia) | Actigraph (<100 count/min) | 0.67 | -3.3 [-9.7 3.1] | -- | [61] |
| CHAMPS | 1.2.2.1/2.3/3.2/4.5 | 870 | OA (USA) | Actigraph (<100 count/min) | 0.72 | -6.8 [-10.6 2.4] | -- | [15] |
| | | 58 | OA (USA) | Actigraph (<100 count/min) | 0.72 | -5.2 [not reported] | -- | [62] |

For peer review only



For peer review only - http://bmjopen.bmj.com/site/about/guidelines.xhtml

| | | | | | | | | |
|---|---|---|---|---|---|---|---|---|
| LASA | 1.2.2.1/2.4/3.1/4.3 | 83 | OA (Netherlands) | Actigraph (<100 count/min) | 0.78 | + 0.2 for 10 item<br>-2.1 [-7.4 3.3] for 6 item | -- | [33] |
| STAQ | | 88 | A (France) | Actigraph (<150 count/min) | 0.72 | -2.4 [-6.2 4.9] | -- | [50] |
| PAST | 1.2.2.1/2.1/3.1/4.5 | 90 | A (Australia) | activPAL (sitting/lying postures) | 0.72 | -1.0 [- 5.6 3.8] | t-test was inconclusive | [42] |
| PAST-U | | 57 | A (Australia) | activPAL (sitting/lying postures) | 0.78 | 0.1 [-3.9 4.1] | -- | [43] |
| SIT-Q-7d | 1.2.2.1/2.2/3.1/4.3 | 51<br><br>402 | A (Belgium)<br>A (UK) | activPAL (sitting/lying postures)<br>actiHeart (<1.5MET) | 0.72 | 1.0 [-4.8 8.2] for Belgian sample<br><br>0.4 [-6.9 8.6] for UK sample | -- | [48] |
| | | 33 & 33 | A & OA (Belgium) | activPAL (sitting/lying postures) | 0.83 | 2.3 [only reported as a %]<br>0.3 [-8.9 0.7] for older adults | -- | [63] |
| | | 442 | OA (Belgium) | Actigraph (<100 count/min) | 0.83 | 1.36 [-6.0 3.3] | -- | [64] |
| MOST | 1.2.2.1/2.2/3.2/4.5 | 48 | OA (Australia) | Actigraph (<100 count/min) | 0.67 | -3.6 [-7.4 -0.2] | Guyatt Index 0.39 (0.47 for Actigraph) | [35] |

A: adults; N: number of participants; OA: older adults; Ref: reference; UK: United Kingdom; USA: United States of America. For tool acronyms see table 1.

For peer review only

Criterion measure

None of the studies tested the accuracy of the tool against direct observation. Only five studies [16,42,43,48,63] used a postural sensor that actually measures sitting time objectively (activPAL), the other seventeen used an accelerometer built to measure low movement as a criterion measure (ActiGraph, actiHeart).

Statistical analysis

Accuracy and Limits of Agreement were usually derived from Bland and Altman plots. Sensitivity to change was defined differently in the two articles which reported this measurement characteristic; one used t-test statistics [42], one used the Guyatt Index [35].

Study Quality

Studies which scored highly for quality tended to be purposefully designed to test measurement characteristics, rather than secondary analysis of data collected for another purpose. The most common loss of quality was due to the use of accelerometers which assess low movement (e.g. ActiGraph) as a criterion measure, as this does not measure the primary aspect of the definition of SB (i.e. posture). Another issue which lowered quality was the manipulation of the criterion measure without clear justification. For example, some studies manipulated the count threshold (used to define SB) or included only SB bouts longer than a particular duration without justification or solid rationale.

Tools and measurement characteristics

For peer review only

Table 3 summarises the results reported by these studies, arranged per measurement tool and mapped against the relevant taxon. Very few of the existing tools to measure SB using self-report have actually been investigated for these measurement characteristics. Accuracy has been reported for ten out of the 37 tools identified in the inventory (IPAQ-L l7d, IPAQ-S l7d, GPAQ, MOST, CHAMPS, LASA, PAST, PAST-U, STAQ, SIT-Q-7d). The most tested tools were the IPAQ in its long form, last seven days [16, 51-55] and short form, last seven days.[56-60]. The SIT-Q-7d was tested in three studies [48, 63-64], and the CHAMPS was investigated in two studies [15, 62]. Information for other tools; GPAQ [61], LASA [33], MOST [35], PAST [42], PAST-U [43], and STAQ [50], come from single studies. Reports of sensitivity to change are only available for two tools; MOST [35] and PAST [42].

Taxa tested

The literature provides measurement characteristics information for eight distinct full taxa:

1.1.1/2.2/3.1/4.3 with six studies on IPAQ-L uw;

1.1.1/2.2/3.1/4.1 with five studies on IPAQ-S uw;

1.1.1/2.4/3.1/4.5 with one study on GPAQ;

1.2.2.1/2.1/3.1/4.5 with one study on PAST and one study on PAST-U;

1.2.2.1/2.2/3.1/4.3 with three studies on SIT-Q-7d;

1.2.2.1/2.2/3.2/4.5 with one study on MOST;

1.2.2.1/2.3/3.2/4.5 with two studies on CHAMPS; and

1.2.2.1/2.4/3.1/4.3 with one study on LASA and one study on STAQ.

For peer review only

For the assessment type, there is information for direct measures via single item (1.1.1, twelve studies) and for composite sums of behaviours (1.2.2.1, ten studies). However, there is no information for direct proxy measures (1.1.2). For recall period, there is information on all four possible categories (2.1 previous day, two studies; 2.2 previous week, sixteen studies; 2.3 longer, two studies; and 2.4 unanchored, two studies). The unanchored recall period (2.4), used by 40% of the tools in the inventory, is particularly under-represented with only two studies in the validation review. For temporal scale there is mostly information for assessment at day scale (3.1, twenty studies) and only three studies for the temporal scale of a week (3.2). This is broadly representative of usage by tools in the inventory. For assessment period there is information for weekdays only (4.1, five studies) or both weekdays and weekend days (4.3, eleven studies) and for tools with the assessment period not defined (4.5, six studies). The assessment period not defined taxon (4.5), used by over half the tools in the inventory, is under-represented by these validation studies.

Accuracy

Information for taxon 1.1.1/2.2/3.1/4.3 (IPAQ-L-l7d) is not equivocal. The majority of studies reported a large underestimation of total SB time ranging from 1.1 hours in adults [54] to 6 hours in older adults [51]. One study reported that tools in this taxon overestimate total SB time by 2.2 hours in adults [53]. It is clear that the systematic error on estimates of total SB time using tools from this taxon is likely to be very large (several hours/day). The random error is also likely to be very large as the Limits of Agreement reported were consistently very large. Information for taxon 1.1.1/2.2/3.1/4.1 (IPAQ-S-l7d) is a little more consistent for adults. Tools in this taxon seem to underestimate total SB time by 1.5 to 3 hours in adults. However, in older adults this was less clear with reports of underestimation by 4.4 hours [59] and overestimation by 3.5 hours [56]. In both populations the error and Limits of Agreement were large, but not as large as for the previous taxon.

For peer review only - http://bmjopen.bmj.com/site/about/guidelines.xhtml

Although not entirely consistent, tools reporting information from a single item as a direct measure of sitting (taxon 1.1.1) tended to underestimate sitting, with underestimation ranging from -0.5 [60] to -6.0 [51] hours per day. Within those tools, the IPAQ-S-l7d (reporting only for week days in the past week, taxa 2.2 & 4.1), tended to have better agreement than the IPAQ-L-l7d (reporting for both week and weekend days in the past week, taxa 2.2 & 4.3), and the GPAQ (reporting over a longer recall period with the assessment period not defined, taxa 2.4 & 4.5). Tools reporting on a sum of behaviours (taxon 1.2.2.1), were more likely to overestimate sitting than for the single item direct measure (taxon 1.1.1). Tools which reported on a sum of behaviours over the past day or past week (taxa 1.2.2.1 & 2.1 or 2.2), tended to have the closest agreement with objective criterion measures, with most studies reporting agreement between -1.0 and +2.3 hours per day. Tools which reported sum of behaviours over a longer (taxon 2.3) or unanchored (taxon 2.4) recall period or which had a temporal unit of a week (taxon 3.2) reported larger underestimates (-2.1 to -6.8 hours/day). In particular the CHAMPS tool, reporting both for a recall period of a year (taxon 2.3) with a temporal unit of a week (taxon 3.2), had the largest differences for any tool. However, there were only a few studies reporting on these aspects, and such conclusions are necessarily tentative. Regardless of level of agreement, limits of agreement were large for all tools.

Sensitivity to change

There is almost no information about sensitivity to change. The two studies that assessed sensitivity to change [35, 42] provided little tangible information. The results were either inconclusive [42], or reported the Guyatt index against a criterion measure which does not measure sitting [35]. While the latter provided some indication that the tools' sensitivity to change was similar to that of an objective measure of low movement it does not give a clear indication as to whether it is sensitive to

For peer review only - http://bmjopen.bmj.com/site/about/guidelines.xhtml

a change in total SB time. Neither of these studies reported the minimal detectable change [65], a metric which provides an easily interpretable value of the capacity of a tool to detect a change.

**DISCUSSION**

A taxonomy (TASST) for the systematic description and comparison of self-reported measures of SB has been established. TASST provides a rigorous framework for informed choice, development and evaluation of self-report tools. This framework has been used to review the measurement characteristics of existing tools in order to identify the optimum tool for population surveillance. The available evidence about measurement characteristics essential for population surveillance, namely accuracy and responsiveness to change, was insufficient to ascertain which tool currently used in practice is best. Accuracy was poor for all existing tools, with both under and over estimation of total time spent in SB and large limits of agreement. In addition, there is a complete lack of evidence about their sensitivity to change. Mapping available evidence onto the TASST framework has enabled informed recommendations to be made about the promising features for a surveillance tool, and identification of the aspects on which future research and development of SB surveillance tools should focus.

The use of a coherent and robust taxonomy (TASST) to systematically evaluate and compare the characteristics of measurement tools is the main strength of this study. However, in terms of accuracy and sensitivity to change, the current published evidence does not cover the entire taxonomy. Consequently, at present, only tentative recommendations can be provided. The taxonomy can be used, however, to identify gaps in current research and provide focussed guidance for future research and development. During the development of TASST, self-report tools which aimed to measure SB in specific populations (e.g. children, those with arthritis) or specialised

For peer review only - http://bmjopen.bmj.com/site/about/guidelines.xhtml



For peer review only

contexts (e.g. workplace) were not considered. However, TASST is a generic framework, so tools specific to these populations may already be fully described by the taxonomy. For example, a question asking about time spent sitting at school which is specific to children, would be covered under the sub-division of the day assessment period (taxon 4.4). Another consequence of the exclusion criteria is that evidence on accuracy and sensitivity to change of tools specific to these populations was not mapped on the taxonomy. Therefore, the conclusions drawn from the measurement characteristics in this study are only valid for adults and older adults. Future research should be conducted using the TASST taxonomy to map existing self-report tools covering those populations and contexts currently excluded from taxonomy development (such as children, schools or the workplace) to identify areas for development. In addition, this study has the general limitations common to most systematic reviews, i.e. included articles were restricted to those written in English, articles and tools published after the date of search were not included, and any relevant articles not identified during the search will have been excluded.

The current study is the first to clearly define and focus on the measurement characteristics required for population surveillance (accuracy and sensitivity to change). There is only one other systematic review reporting on the measurement characteristics of self-report tools to measure SB [13], which concentrated on validity (assessed through rank correlation) and reliability, which are the measurement characteristics relevant to establishing associations between SB and health. In agreement with the previous review, we found that the major flaw of most validation studies was the use of an inadequate criterion measure. The choice of criterion measure depends on the purpose of the tool. While direct observation should be considered the gold standard, if the purpose is to assess total sedentary time, then accurate postural sensors should be adequate (e.g. activPAL). In this review, only five out of 22 studies used an adequate criterion measure. Instead, many studies used an accelerometer which measures low levels of movement at the hip (e.g. ActiGraph) as a

For peer review only - http://bmjopen.bmj.com/site/about/guidelines.xhtml

criterion measure, but such tools do not measure SB directly and can misclassify standing as sitting [12]. Although it is possible that criterion measure may have provided a confounding effect on agreement (e.g. tools assessing previous day recall period (taxon 2.1, PAST, PAST-U) were only assessed against the activPAL), no clear trend towards better or worse agreement with a particular type of criterion measure or ActiGraph cut-off was apparent.

Despite the incomplete nature of the evidence, TASST enables the identification of desirable characteristics of self-report tools to measure SB when used for population surveillance. Firstly, tools assessing total SB time as a sum of behaviours (taxon 1.2.2.1; CHAMPS, LASA, MOST, PAST, PAST-U, SIT-Q-7d, STAQ) provided better accuracy than single item direct measurement (taxon 1.1.1; IPAQ-L-l7d, IPAQ-S-l7d and GPAQ) tools, especially when comparing tools with equivalent recall periods. However, this will be dependent on the behaviours or domains included within the sum, and whether they are exhaustive, consistent and mutually exclusive. Tools with a non-exhaustive sum will underestimate total time, for example, the Longitudinal Aging Study Amsterdam (LASA), found that a six item sum provided a better correlation with SB across the sample, but that a ten item sum was more accurate [33]. Conversely, tools which contain behaviours which might occur concurrently (such as watching TV and using a tablet computer) may lead to an over-estimate in total SB time [63]. Secondly, tools using a previous day recall period (taxon 2.1, PAST, PAST-U) tended to provide better accuracy than those with longer recall periods (taxa 2.2, 2.3 and 2.4). This corroborates recent research on the validity of computerised survey systems which assess SB using a past-day recall period [17, 66]. However, although tools using previous day recall may more accurate, it is likely that their sensitivity to change will be less good due to the higher underlying variability in daily SB [67].

For peer review only

Most tools currently used for population surveillance of SB systematically underestimate the amount of SB by two to four hours per day. Yet, self-report tools are still the most practical and economical means of population surveillance. Therefore, policy makers and clinicians should be aware that reports of population SB time are likely to be grossly underestimated, and should be cognisant of this fact when making decisions on implementing, developing and evaluating policy and public health interventions. In addition, policy makers and clinicians should be cautious in interpreting any reported difference in population SB time as a real change. The dearth of information about sensitivity to change of these tools means that we do not know the magnitude of change required to be certain that a change is real and not background variation. Moving forward, development of national and international surveillance systems should not be undertaken assuming that a tool is adequate because it has been used previously. Instead, investment should be made in research to evaluate the sensitivity to change and accuracy of tools to measure SB, paying attention to the potential trade-off between these two measurement characteristics. Such research should be carefully planned, to ensure that meaningful comparisons are investigated. The TASST taxonomy should be used as a useful framework to facilitate such a systematic approach.

For peer review only

Acknowledgements

The named authors present the study on behalf of the Seniors USP Team, which comprises: Dawn A Skelton (PI), Sebastien Chastin, Simon Cox, Elaine Coulter, Iva Čukić, Philippa Dall, Ian Deary, Geoff Der, Manon Dontje, Claire Fitzsimons, Catherine Gale, Jason Gill, Malcolm Granat, Cindy Gray, Carolyn Greig, Elaine Hindle, Karen Laird, Gillian Mead, Nanette Mutrie, Victoria Palmer, Ratko Radakovic, Naveed Sattar, Richard Shaw, John Starr, Sally Stewart, Sally Wyke. The authors would like to thank Calum Leask (CL) for his help in updating both of the searches in November 2016.

Competing Interests:

All authors had financial support from the Medical Research Council for the submitted work; no financial relationships with any organisations that might have an interest in the submitted work in the previous three years; no other relationships or activities that could appear to have influenced the submitted work.

Funding:

The study was performed in preparation for the Seniors USP: Understanding Sedentary Patterns project, which was supported by the Medical Research Council [MR/K025023/1]

Authors Contributions:

SC and DS conceived and supervised the study. PD and SC designed the study. PD, EC, CF and SC collected the data, and performed the systematic review. PD, CF and SC analysed the data for the

For peer review only

taxonomy. PD and SC analysed the data from the systematic review. All authors interpreted the data and critically revised the manuscript for important intellectual content.

Data Sharing: Data can be obtained from the corresponding author on request.

Figure Legends

Figure 1: TAxonomy of Self-reported Sedentary behaviour Tools (TASST)

Figure 2: PRISMA diagram of the validation systematic review

For peer review only

## References

1. Kohl HW 3rd, Craig CL, Lambert EV, et al. The pandemic of physical inactivity: global action for public health. *Lancet* 2012;380:294–305.

2. Chastin S, Scwartz U, Skelton D. Development of a consensus taxonomy of sedentary behaviors (SIT): report of Delphi round 1. *PLoS One* 2013;8:e82313.

3. Sedentary Behaviour Research Network. Standardized use of the terms “sedentary” and “sedentary behaviours”. *Appl Physiol Nutr Metabol* 2012;37:540-2.

4. Healy GN, Matthews CE, Dunstan DW, Winkler EAH, Owen N. Sedentary time and cardio-metabolic biomarkers in US adults: NHANES 2003-06. *Eur Heart J* 2011;32:590-7.

5. Harvey JA, Chastin SFM, Skelton DA. How sedentary are older people? A systematic review of the amount of sedentary behavior. *J Aging Phys Act* 2015;23:471-87.

6. Proper KI, Singh AS, van Mechelen W, Chinapaw MJM. Sedentary behaviors and health outcomes among adults: a systematic review of prospective studies. *Am J Prev Med* 2011,40:174-82.

7. Thorp AA, Owen N, Neuhaus M, Dunstan DW. Sedentary behaviors and subsequent health outcomes in adults a systematic review of longitudinal studies, 1996-2011. *Am J Prev Med* 2011;41:207-15.

8. de Rezende LFM, Rey-López JP, Matsudo VKR, do Carmo Luiz O. Sedentary behavior and health outcomes among older adults: a systematic review. *BMC Public Health* 2014;14:333.

9. Dogra S, Stathokostas L. Sedentary behavior and physical activity are independent predictors of successful aging in middle-aged and older adults. *J Aging Res* 2012;2012:190654.

For peer review only

10. Department of Health. Start Active, Stay Active: A report on physical activity from the four home countries' Chief Medical Officers. 2011.

11. Guyatt G, Walter S, Norman G. Measuring change over time. assessing the usefulness of evaluative instruments. *J Chron Dis* 1987;40:171-8.

12. Kozey-Keadle S, Libertine A, Lyden K, Staudenmayer J, Freedson P. Validation of wearable monitors for assessing sedentary behavior. *Med Sci Sports Exerc* 2011;43:1561-7.

13. Healy GN, Clark BK, Winkler EAH, Gardiner PA, Brown WJ, Matthews CE. Measurement of adults' sedentary time in population-based studies. *Am J Prev Med* 2011,;41:216-27.

14. Bauman A, Ainsworth BE, Sallis JF, et al. The descriptive epidemiology of sitting: a 20 country comparison using the International Physical Activity Questionnaire (IPAQ). *Am J Prev Med* 2011;41:228-35.

15. Hekler EB, Buman MP, Haskell WL, et al. Reliability and validity of CHAMPS self-reported sedentary-vigorous intensity physical activity in older adults. *J Phys Act Health* 2012;9:225-36.

16. Chastin SFM, Culhane B, Dall PM. Questionnaire sitting time (IPAQ) compared to objective measurement via inclinometry (activPAL). *Physiol Meas* 2014;35:2319-28.

17. Kozey-Keadle S, Lyden K, Hickey A, et al. Validation of a previous day recall for measuring the location and purpose of active and sedentary behaviours compared to direct observation. *Int J Behav Nutr Phys Act* 2014;11:12.

18. Kmet LM, Lee RC, Cook LS. Standard quality assessment criteria for evaluating primary research papers from a variety of fields. *Edmonton:* Alberta Heritage Foundation for Medical Research 2004;1-22.

19. Banks E, Jorm L, Rogers K, Clements M, Bauman A. Screen-time, obesity, ageing and disability: findings from 91 266 participants in the 45 and Up Study. *Public Health Nutr* 2011;14:34-43.

For peer review only

20 Patel AV, Bernstein L, Deka A, et al. Leisure time spent sitting in relation to total mortality in a prospective cohort of US adults. *Am J Epidemiol* 2010;172:419-429.

21. Salmon J, Owen N, Crawford D, Bauman A, Sallis JF. Physical activity and sedentary behavior: a population-based study of barriers, enjoyment, and preference. *Health Psychol* 2003;22:178-88.

22. Gardiner PA, Healy GN, Eakin EG, et al. Associations between television viewing time and overall sitting time with the metabolic syndrome in older men and women: The Australian Diabetes Obesity and Lifestyle study. *JAGS* 2011;59:788-96.

23. Shields M, Tremblay MS. Sedentary behaviour and obesity. *Health Rep.* 2008;19:19-30.

24. Katzmarzyk PT, Church TS, Craig CI, Bouchard C. Sitting time and mortality from all causes, cardiovascular disease, and cancer. *Med Sci Sports Exerc* 2009;41:998-1005.

25. Hamer M, Stamatakis E. Screen-based sedentary behaviour, physical activity, and muscle strength in the English Longitudinal Study of Ageing. *PLoS One* 2013;8:e66222.

26. Wareham NJ, Jakes RW, Rennie KL, Mitchell J, Hennings S, Day NE. Validity and repeatability of the EPIC-Norfolk physical activity questionnaire. *Int J Epidemiol* 2002;31:186-74.

27. Parsons TJ, Thomas C, Power C. Estimated activity patterns in British 45 year olds: cross-sectional findings from the 1958 British birth cohort. *Eur J Clin Nutr* 2009;63:978-85.

28. Armstrong T, Bull F. Development of the World Health Organization Global Physical Activity Questionnaire (GPAQ). *J Public Health* 2006;14:66-70.

29. Stamatakis E, Davis M, Stathi A, Hamer M. Associations between multiple indicators of objectively-measured and self-reported sedentary behaviour and cardiometabolic risk in older adults. *Prev Med* 2012;54:82-7.

For peer review only

567 30. Chau JY, Grunseit A, Midthjell K, et al. Sedentary behaviour and risk of mortality from all-causes and cardiometabolic diseases in adults: evidence from the HUNT3 population cohort. *Br J Sports Med* 2015;49:737-42.

570 31. Craig CL, Marshall AL, Sjöström M, et al. International Physical Activity Questionnaire: 12-country reliability and validity. *Med Sci Sports Exerc* 2003;35:1381-95.

572 32. Proper KI, Cerin E, Brown WJ, Owen N. Sitting time and socio-economic differences in overweight and obesity. *Int J Obes* 2007;31:169-76.

574 33. Visser M, Koster A. Development of a questionnaire to assess sedentary time in older persons – a comparative study using accelerometry. *BMC Geriatr* 2013;13:80.

576 34. Burazeri G, Goda A, Kark JD. Television viewing, leisure-time exercise and acute coronary syndrome in transitional Albania. *Prev Med* 2008;47:112-5.

578 35. Gardiner PA, Clark BK, Healy GN, Eakin EG, Winkler EAH, Owen N. Measuring older adults' sedentary time: reliability, validity, and responsiveness. *Med Sci Sports Exerc* 2011;43: 2127-33.

580 36. Ford ES. Combined television viewing and computer use and mortality from all-causes and diseases of the circulatory system among adults in the United States. *BMC Public Health* 2012;12:70

582 37. Evenson KR, McGinn AP. Test-retest reliability of adult surveillance measures for physical activity and inactivity. *Am J Prev Med* 2005;28:470-8.

584 38. Wolf AM, Hunter DJ, Colditz GA, et al. Reproducibility and validity of a self-administered physical activity questionnaire. *Int J Epidemiol* 1994;23:991-99.

586 39. Matthews CE, George SM, Moore SC, et al. Amount of time spent in sedentary behaviors and cause-specific mortality in US adults. *Am J Clin Nutr* 2012;ajcn-019620.

For peer review only

40. Salmon J, Bauman A, Crawford D, Timperio A, Owen N. The association between television viewing and overweight among Australian adults participating in varying levels of leisure-time physical activity. *Int J Obes Relat Metab Disord* 2000;24:600-6.

41. Washburn RA, McAuley E, Katula J, Mihalko SL, Boileau RA. The Physical Activity Scale for the Elderly (PASE): evidence for validity. *J Clin Epidemiol* 1999;52:643-51.

42. Clark BK, Winkler E, Healy GN, et al. Adults' past-day recall of sedentary time: reliability, validity and responsiveness. *Med Sci Sports Exerc* 2013;45:1198-207.

43. Clark BK, Pavey TG, Lim RF, Gomersall SR, Brown WJ. Past-day recall of sedentary time: validity of a self-reported measureof sedentary time in a university population. *J Sci Med Sport* 2016;19:237-41.

44. Balboa-Castillo T, León-Muñoz LM, Graciani A, Rodríguez-Artalejo F, Guallar-Castillón P. Longitudinal association of physical activity and sedentary behavior during leisure time with health-related quality of life in community-dwelling older adults. *Health Qual Life Outcomes* 2011;9:41-47.

45. Rosenberg DE, Norman GK, Wagner N, Patrick K, Calfas KJ, Sallis JF. Reliability and validity of the Sedentary Behaviour Questionnaire (SBQ) for adults. *J Phys Act Health* 2010;7:697-705.

46. Stamatakis E, Hamer M, Dunstan DW. Screen-based entertainment time, all-cause mortality, and cardiovascular events. *J Am Coll Cardiol* 2011;57:292-9.

47. Lynch BM, Friedenreich CM, KHandwala F, Liu A, Nicholas J, Csizmadi I. Development and testing of a past year measure of sedentary behaviour: the SIT-Q. *BMC Public Health*; 2014;14:899.

48. Wijndaele K, de Bourdeaudhuij I, Godino JG, et al. Reliability and validity of a domain-specific last 7-d sedentary time questionnaire. *Med Sci Sports Exerc* 2014;46:1248-60.

For peer review only

49. Csizmadi I, Neilson HK, Kopciuk KA, et al. The Sedentary Time and Activity reporting Questionnaire (STAR-Q): reliability and validity against doubly labelled water and 7-day activity diaries. *Am J Epidemiol* 2014;80:424-35.

50. Mensah K, Maire A, Oppert J-M, et al. Assessment of sedentary behaviours and transport-related activities by questionnaire: a validation study. *BMC Public Heath* 2016;16:753.

51. Dahl-Pederson KI, Hansen AW, Bjerregaard P, Jørgensen ME, Brage S. Validity of the international physical activity questionnaire in the arctic. *Med Sci Sports Exerc* 2013;45:728-36.

52. van Dyck D, Cardon G, Deforche B, de Bourdeaudhuij I. IPAQ interview version: convergent validity with accelerometers and comparison of physical activity and sedentary time levels with the self-administered version. *J Sports Med Phys Fitness* 2014;55:776-86.

53. Hagstromer M, Ainsworth BE, Sjostrom M. Comparison of a subjective and an objective measure of physical activity in a population sample. *J Phys Act Health* 2010;7:541-50.

54. Celis-Morales CA, Perez-Bravo F, Ibanez L, Salas C, Bailey MES, Gill JMR. Objective vs. self-reported physical activity and sedentary time: effects of measurement method on relationships with risk biomarkers. *PLoS ONE* 2012;7:e36345.

55. Wanner M, Probst-Hensch N, Kriemler S, Meier F, Autenrieth C, Martin BW. Validation of the long international physical activity questionnaire: influence of age and language region. *Prev Med Rep* 2016;3:250-6.

56. Dyrstad SM, Hansen BH, Holme IM, Anderssen SA. Comparison of self-reported versus accelerometer-measured physical activity. *Med Sci Sports Exerc* 2014;46:99-106.

57. Oyeyemi AL, Umar M, Oguche F, Aliyu SU, Oyeyemi AY. Accelerometer-determined physical activity and its comparison with the International Physical Activity Questionnaire in a sample of Nigerian adults. *PLoS ONE* 2014;9:e87233.

For peer review only

58. Hurtig-Wennlöf A, Hagströmer M, Olsson L. The International Physical Activity Questionnaire modified for the elderly: aspects of validity and feasibility. *Public Health Nutr* 2010;13:1847-54.

59. Grimm EK, Swartz AM, Hart T, Miller NE, Strath SJ. Comparison of the IPAQ-short form and accelerometry predictions of physical activity in older adults. *J Aging Phys Act* 2012;20:64-79.

60. Curry WB, Thompson JL. Comparability of Accelerometer- and IPAQ-derived physical activity and sedentary time in south Asian women: a cross-sectional study. *Eur J Sport Sci* 2015;15:655-62.

61. Alkahtani SA. Convergent validity: agreement between accelerometry and the Global Physical Activty Questionnaire in college-age Saudi men. *BMC Res Notes* 2016;9:436.

62. Gennuso KP, Matthews CE, Colbert LH. Reliability and validity of two self-report measures to assess sedentary behavior in older adults. *J Phys Act Health* 2015;12:727-32.

63. Busschaert C, de Bourdeaudhuij I, van Holle V, Chastin SFM, Cardon G, de Cocker K. Reliability and validity of three questionnaires measuring context-specific sedentary behaviour and associated correlates in adolescents, adults, and older adults. *Int J Behav Nutr Phys Act* 2015;12:117.

64. van Cauwenberg J, van Holle V, de Bourdeaudhuij I, Owen N, Deforche B. Older adults' reporting of specific sedentary behaviours: validity and reliability. *BMC Public Health* 2014;14:734.

65. Beaton DE, Bombardier C, Katz JN, Wright JG. A taxonomy for responsiveness. *J Clin Epidemiol* 2001;54:1204-71.

66. Gomersall SR, Pavey T, Clark BK, Jasman A, Brown WJ. Validity of a self-report recall tool for estimating sedentary behaviour in adults. *J Phys Act Health* 2015;12:1485-91.

67. Hart TL, Swartz AM, Cashin SE, Strath SJ. How many days of monitoring predict physical activity and sedentary behaviour in older adults? *Int J Behav Nutr Phys Act* 2011;8:62.

For peer review only

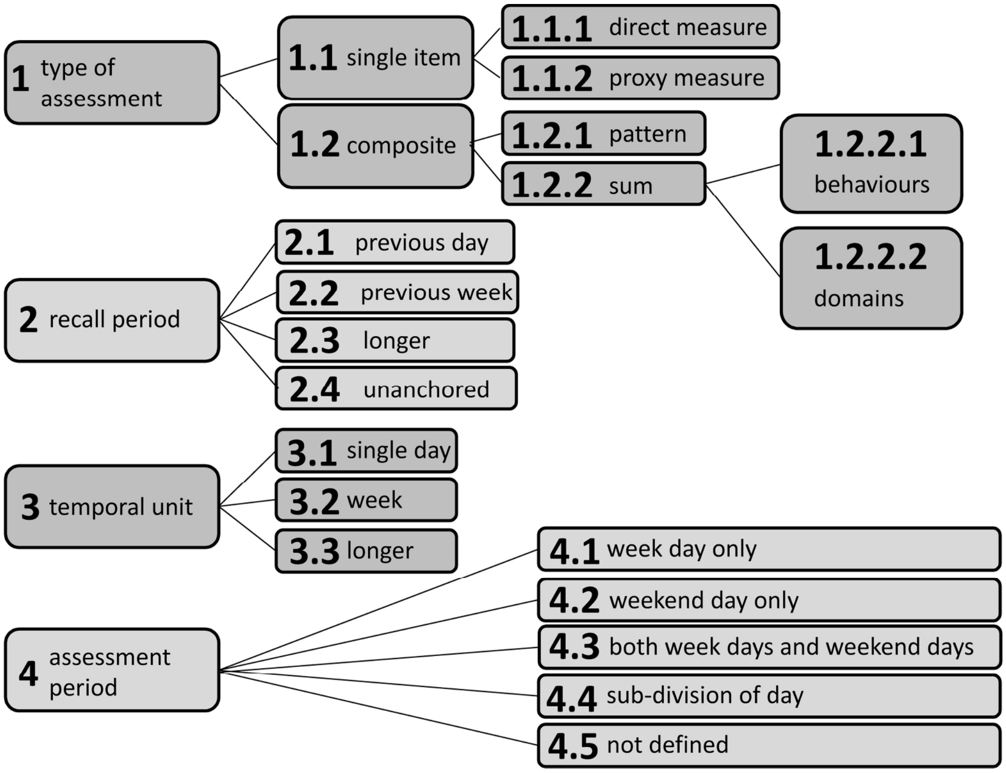


TAxonomy of Self-reported Sedentary behaviour Tools (TASST)
Figure 1
186x142mm (300 x 300 DPI)

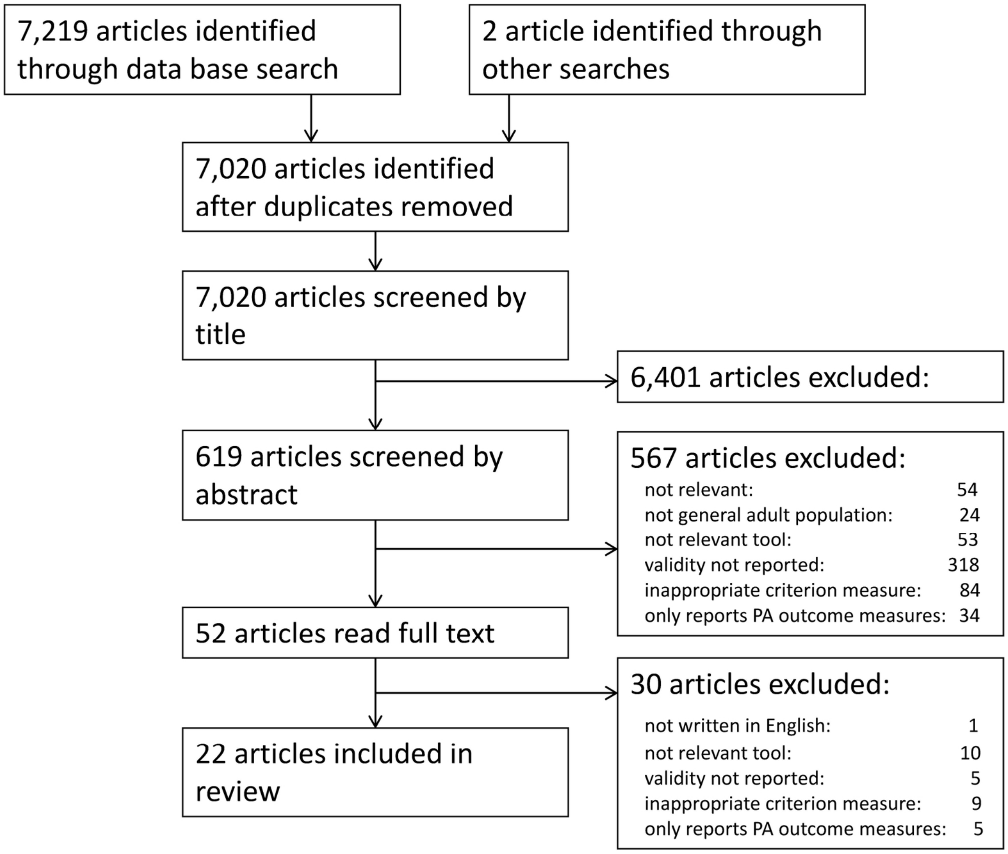


PRISMA diagram of the validation systematic review
Figure 2
171x144mm (300 x 300 DPI)

# PRISMA 2009 Checklist

| Section/topic | # | Checklist item | Reported on page # |
|---|---|---|---|
| **TITLE** | | | |
| Title | 1 | Identify the report as a systematic review, meta-analysis, or both. | 1 |
| **ABSTRACT** | | | |
| Structured summary | 2 | Provide a structured summary including, as applicable: background; objectives; data sources; study eligibility criteria, participants, and interventions; study appraisal and synthesis methods; results; limitations; conclusions and implications of key findings; systematic review registration number. | 2 |
| **INTRODUCTION** | | | |
| Rationale | 3 | Describe the rationale for the review in the context of what is already known. | 3 |
| Objectives | 4 | Provide an explicit statement of questions being addressed with reference to participants, interventions, comparisons, outcomes, and study design (PICOS). | 3 |
| **METHODS** | | | |
| Protocol and registration | 5 | Indicate if a review protocol exists, if and where it can be accessed (e.g., Web address), and, if available, provide registration information including registration number. | 4 |
| Eligibility criteria | 6 | Specify study characteristics (e.g., PICOS, length of follow-up) and report characteristics (e.g., years considered, language, publication status) used as criteria for eligibility, giving rationale. | 4 & 5 |
| Information sources | 7 | Describe all information sources (e.g., databases with dates of coverage, contact with study authors to identify additional studies) in the search and date last searched. | 4 & 5 |
| Search | 8 | Present full electronic search strategy for at least one database, including any limits used, such that it could be repeated. | 5 |
| Study selection | 9 | State the process for selecting studies (i.e., screening, eligibility, included in systematic review, and, if applicable, included in the meta-analysis). | 5 |
| Data collection process | 10 | Describe method of data extraction from reports (e.g., piloted forms, independently, in duplicate) and any processes for obtaining and confirming data from investigators. | 5 |
| Data items | 11 | List and define all variables for which data were sought (e.g., PICOS, funding sources) and any assumptions and simplifications made. | 5 |
| Risk of bias in individual studies | 12 | Describe methods used for assessing risk of bias of individual studies (including specification of whether this was done at the study or outcome level), and how this information is to be used in any data synthesis. | Not reporting on trials, so not done |

For peer review only

# PRISMA 2009 Checklist

| | | | |
|---|---|---|---|
| Summary measures | 13 | State the principal summary measures (e.g., risk ratio, difference in means). | 5 |
| Synthesis of results | 14 | Describe the methods of handling data and combining results of studies, if done, including measures of consistency (e.g., $I^2$) for each meta-analysis. | Not done |



| Section/topic | # | Checklist item | Reported on page # |
|---|---|---|---|
| Risk of bias across studies | 15 | Specify any assessment of risk of bias that may affect the cumulative evidence (e.g., publication bias, selective reporting within studies). | 9 |
| Additional analyses | 16 | Describe methods of additional analyses (e.g., sensitivity or subgroup analyses, meta-regression), if done, indicating which were pre-specified. | Not done |
| **RESULTS** | | | |
| Study selection | 17 | Give numbers of studies screened, assessed for eligibility, and included in the review, with reasons for exclusions at each stage, ideally with a flow diagram. | 8, figure 2 |
| Study characteristics | 18 | For each study, present characteristics for which data were extracted (e.g., study size, PICOS, follow-up period) and provide the citations. | Table 3 |
| Risk of bias within studies | 19 | Present data on risk of bias of each study and, if available, any outcome level assessment (see item 12). | 8 |
| Results of individual studies | 20 | For all outcomes considered (benefits or harms), present, for each study: (a) simple summary data for each intervention group (b) effect estimates and confidence intervals, ideally with a forest plot. | Table 3, pages 9 & 10 (not a review of interventions) |
| Synthesis of results | 21 | Present results of each meta-analysis done, including confidence intervals and measures of consistency. | Not enough data for meta-analysis |
| Risk of bias across studies | 22 | Present results of any assessment of risk of bias across studies (see Item 15). | 9 |
| Additional analysis | 23 | Give results of additional analyses, if done (e.g., sensitivity or subgroup analyses, meta-regression [see Item 16]). | Not done |
| **DISCUSSION** | | | |
| Summary of evidence | 24 | Summarize the main findings including the strength of evidence for each main outcome; consider their relevance to key groups (e.g., healthcare providers, users, and policy makers). | 12 |
| Limitations | 25 | Discuss limitations at study and outcome level (e.g., risk of bias), and at review-level (e.g., incomplete retrieval of identified research, reporting bias). | 11 & 12 |

For peer review only

# PRISMA 2009 Checklist

| | | | |
|---|---|---|---|
| Conclusions | 26 | Provide a general interpretation of the results in the context of other evidence, and implications for future research. | 12 |
| **FUNDING** | | | |
| Funding | 27 | Describe sources of funding for the systematic review and other support (e.g., supply of data); role of funders for the systematic review. | 14 |

*From:* Moher D, Liberati A, Tetzlaff J, Altman DG, The PRISMA Group (2009). Preferred Reporting Items for Systematic Reviews and Meta-Analyses: The PRISMA Statement. PLoS Med 6(6): e1000097. doi:10.1371/journal.pmed1000097

For more information, visit: www.prisma-statement.org.

Page 2 of 2

For peer review only
